# Design of experiment (DoE)-driven *in vitro* and *in vivo* uptake studies of exosomes for pancreatic cancer delivery enabled by copper-free click chemistry-based labelling

**DOI:** 10.1080/20013078.2020.1779458

**Published:** 2020-06-19

**Authors:** Lizhou Xu, Farid N. Faruqu, Revadee Liam-or, Omar Abu Abed, Danyang Li, Kerrie Venner, Rachel J Errington, Huw Summers, Julie Tzu-Wen Wang, Khuloud T. Al-Jamal

**Affiliations:** a School of Cancer & Pharmaceutical Sciences, Faculty of Life Sciences & Medicine, King’s College London, London, UK; b Health Science Department, Faculty of Graduate Studies, Arab American University in Palestine, Ramallah, Palestine; c Institute of Neurology, University College London, London, UK; d Division of Cancer and Genetics, School of Medicine, Cardiff University, Cardiff, UK; e College of Engineering, Swansea University, Crymlyn Burrows Swansea, UK

**Keywords:** Exosome, pancreatic cancer, dosimetry, cellular uptake, surface labelling, DoE

## Abstract

Exosomes (Exo)-based therapy holds promise for treatment of lethal pancreatic cancer (PC). Limited understanding of key factors affecting Exo uptake in PC cells restricts better design of Exo-based therapy. This work aims to study the uptake properties of different Exo by PC cells. Exo from pancreatic carcinoma, melanoma and non-cancer cell lines were isolated and characterised for yield, size, morphology and exosomal marker expression. Isolated Exo were fluorescently labelled using a novel in-house developed method based on copper-free click chemistry to enable intracellular tracking and uptake quantification in cells. Important factors influencing Exo uptake were initially predicted by Design of Experiments (DoE) approach to facilitate subsequent actual experimental investigations. Uptake of all Exo types by PC cells (PANC-1) showed time- and dose-dependence as predicted by the DoE model. PANC-1 cell-derived exosomes (PANC-1 Exo) showed significantly higher uptake in PANC-1 cells than that of other Exo types at the longest incubation time and highest Exo dose. *In vivo* biodistribution studies in subcutaneous tumour-bearing mice similarly showed favoured accumulation of PANC-1 Exo in self-tissue (i.e. PANC-1 tumour mass) over the more vascularised melanoma (B16-F10) tumours, suggesting intrinsic tropism of PC-derived Exo for their parent cells. This study provides a simple, universal and reliable surface modification approach *via* click chemistry for *in vitro* and *in vivo* exosome uptake studies and can serve as a basis for a rationalised design approach for pre-clinical Exo cancer therapies.

## Introduction

Pancreatic cancer (PC) is one of the most devastating cancer, of which its mortality rate is the highest among all cancer types owing to long asymptomatic disease progression and poor early detection [1,2]. Development of promising therapeutic tools is needed for the treatment of PC. Recent research interests are drawn towards exosomes, which are 50–150 nm multivesicular body-derived extracellular vesicles released by various cells and are present in biological fluids or *in vitro* cell culture supernatants [3]. Exosomes possess the ability to deliver their cargoes, e.g. proteins, lipids, and nucleic acids to distant recipient cells and these cargoes can induce changes in recipient cells related to regular physiological functioning or pathological progression [4]. There are an increasing number of reports demonstrating the potential of using exosomes as nanocarriers for improved delivery of exogenously loaded drug therapeutics as novel treatment strategies for PC [5]. For example, exosomes were used to deliver siRNA to oncogenic K-RasG12D, a common mutation in PC, resulting in suppression of PC and increasing overall survival in mice [5]. Curcumin was also reported to be delivered by exosomes to PC cells, resulting in anti-inflammatory effect and a significant reduction of pancreatic adenocarcinoma cell viability [1]. It has been reported that exosomes show better uptake profiles in mouse models as compared to liposomes, potentially due to the unique set of proteins (e.g. various integrins, adhesion proteins and phosphatidylserine) present on exosomal membranes which play important roles in facilitating uptake [6,7,8]. Certain exosomes were also reported to express a transmembrane protein called CD47 which can protect them from phagocytosis and result in prolonged *in vivo* circulation time [5]. This naturally occurring factor, therefore, provides similar advantages to that by PEGylation of other synthetic nanoparticles without the drawback of reduced cellular uptake associated with the latter [9].

Despite various attempts, progress in exosome-mediated cancer therapies including PC remained slow. This is largely due to the limited understanding of exosome-cell interaction. Recipient cells were reported to internalise exosomes by a variety of mechanisms such as receptor-mediated pathways, macropinocytosis, phagocytosis and membrane fusion [10–13]. Various cells have been demonstrated to take up exosomes from different cell sources, but to different extents [14,15]. Non-biological factors such as incubation time of cells with exosome and exosome dose were reported to affect cellular uptake of exosomes [16,17]. Interestingly, it was reported that exosomes potentially have tropism towards their cell/tissue of origin [18], and that tumour cell lines were reported to show higher uptake of tumour-derived exosomes compared to non-cancer immortalised cell lines [17]. Preferential uptake of tumour-derived exosomes by their parent cells was demonstrated *in vitro* and *in vivo* in an ovarian cancer model [19]. However, a systematic study investigating the significance of non-biological factors such as incubation time and exosome dose, as well as the tropism of exosomes for their parent cells in PC models is currently unavailable.

Fluorescence labelling of exosomes can facilitate the investigation of their *in vitro* and *in vivo* uptake. Current approaches are mostly based on non-covalent fluorescence labelling strategies involving the use of lipophilic dyes (e.g. PKH26, PKH67, DiI and DiO). Such labelling methods are associated with drawbacks such as aggregation or micelles formation in aqueous solutions, dye leakage and non-specific exchange with endogenous tissue membranes [18,20–23]. These result in false-positive signals such as non-exosome-associated dye-positive particles indistinguishable from labelled exosomes, leading to data misinterpretations [24]. Therefore, a reliable and efficient exosome fluorescent labelling approach is crucial for accurate interpretation of the results from their uptake studies.

This work aims to study the important factors governing uptake of exosomes sourced from different cell types in *in vitro* and *in vivo* PC models for a rational selection of unmodified exosomes as nanocarriers for delivery of therapeutics to PC. This is facilitated by a novel, universal and reliable exosome surface fluorescence labelling approach based on copper-free click chemistry. Design of Experiments (DoE) was employed as a modelling platform to predict the significance of multiple parameters (i.e. incubation time, exosome dose and exosome-parent cell pairing) and their multifactorial interactions in exosome cellular uptake. The DoE predictive model was then validated using semi-quantitative uptake studies of exosomes from the different cell sources by PC cells *in vitro* and *in vivo*.

## Materials and methods

### Cell culture

B16-F10 (ATCC CRL-6475) and PANC-1 (ATCC CRL-1469) cell lines were grown in Advanced RPMI 1640 media, and HEK-293 cell line (ATCC CRL-1573) was grown in MEM media, both supplemented with 10% exosome-free FBS, 1% Penicillin-Streptomycin and 1% GlutaMAX^TM^ (Gibco, Thermo Fisher Scientific). HPAC cell line (ATCC CRL-2119) was grown in DMEM/F12 (1:1) media, supplemented with 10% exosome-free FBS and 1% Penicillin-Streptomycin. All cells were maintained at 37°C in a humidified incubator containing 5% CO_2_. Exosome-depleted FBS was prepared by subjecting FBS to ultracentrifugation (Optima XE, Beckman Coulter) at 100,000 g for 18 h at 4°C. The FBS supernatant post-centrifugation was collected and sterile-filtered using 0.22 µm filters for use in cell culture.

### Exosome isolation

Culture supernatants of B16-F10, PANC-1 and HEK-293 cells were harvested after 1-week culture in CELLine AD1000 bioreactor flasks (WHEATON UK). Cells from 4 × T75 flasks (80% confluent) in 15 mL medium supplemented with 10% exosome-depleted FBS were seeded into the cell compartment of 1 bioreactor flask. The medium reservoir compartment of the flask was filled with 500 mL of the medium supplemented with 10% normal FBS. Culture supernatant or conditioned medium (CM) was harvested from the cell compartment of the flask on a weekly basis and replaced with 15 mL of fresh medium supplemented with 10% exosome-depleted FBS. To isolate exosomes, cell debris was firstly removed by centrifugation at 400 × g for 7 min and at 2,000 × g for 15 min at 4°C. The supernatant was filtered through a 0.22 µm filter (Millipore). Exosomes were then isolated by ultracentrifugation onto a sucrose cushion (25% w/w sucrose in D_2_O, density 1.18–1.20 g/mL) at 100,000 × g for 90 min at 4°C. Upon completion, the sucrose solution layer was collected, washed with PBS, and centrifuged at 100,000 × g for 90 min at 4°C. The final pellet containing exosomes was resuspended in 200 µL sterile PBS and aliquoted before storage at −80°C.

### Exosome characterisation

#### Size distribution and particle concentration of exosomes

The size distribution and particle number/concentration of exosomes were measured by nanoparticle tracking analysis (NTA) using a Nanosight LM10 system with blue (488 nm) laser (Malvern Instruments, UK). Exosomes were diluted in filtered deionised water to obtain 20–60 vesicles per field of view for optimal tracking. Three videos of 30 s were taken and analysed using the NanoSight NTA 3.2 software. The area under the histogram for each triplicate measurement was averaged and used as one particle concentration measurement. All NTA measurements were done with identical system settings for consistency.

#### Exosomal surface proteins characterisation using flow cytometry

Exosome suspension in PBS (40 µL) was mixed with 10 µL of beads (aldehyde sulphate latex 4% w/v, Thermo Fisher Scientific, UK) for 15 min at room temperature (RT). PBS only was used as control. Bovine serum albumin (BSA, 100 µM, 5 µL, Thermo Fisher Scientific, UK) was added into the exosome-bead mixture and incubated for 15 min at RT. One millilitre of PBS was then added and incubated for another 75 min at RT under gentle mixing. The beads were pelleted by centrifugation at 580 × g for 5 min. After supernatant removal, the pellet was incubated in 1 mL of 100 mM glycine for 30 min at RT. The beads were washed with 1 mL of PBS twice, and resuspended in 150 µL of PBS. Exo-bead complex was stained with anti-CD9 (clone HI9a (anti-human), clone MZ3 (anti-mouse), BioLegend, UK), anti-CD63 (clone TS63 (anti-human), clone EPR21151 (anti-mouse), Abcam, UK) or anti-CD81 (clone 5A6 (anti-human), clone Eat-2 (anti-mouse), BioLegend, UK) primary antibodies (anti-human for PANC-1 and HEK-293 exosomes; anti-mouse for B16-F10 exosomes) for 45 min at 4°C, and then stained with the Cy5-conjugated secondary antibody (eBioscience, UK) for 30 min at RT. The pellets were washed and resuspended with an appropriate volume of 3% FBS/PBS for flow cytometry (BD FACSCalibur^TM^, USA). A total of 50,000 events were collected and the mean fluorescence intensity (MFI) was recorded. Degree of expression of the markers is expressed as the fold increase in MFI values to Exo-beads complex stained with the Cy5-conjugated secondary antibody only.

#### Protein amount quantification

Protein amounts in exosome and cell lysates were quantified using micro BCA and regular BCA kits (Thermo Fisher Scientific, UK), respectively, following the protocol provided by the supplier. For cell lysate preparation, cells were cultured in 75 cm^2^ flasks until 80–90% confluent. Cells were then detached with trypsin, neutralised with culture media and pelleted by spinning at 400 × g for 5 min. The supernatant was discarded, and the cells were washed with PBS twice using the same centrifugation conditions as above, before the addition of lysis buffer (RIPA buffer with protease inhibitor cocktail added) (Merck, UK) to the cell pellet from the final spin. The cells were kept on ice for 30 min, vortexing every 10 min to ensure maximal lysis. The mixture was then subjected to centrifugation at 20,000 × g for 30 min at 4°C, and the supernatant (i.e. the lysate) was collected in fresh microcentrifuge tubes, and kept at −80°C until use.

#### Luminal exosomal protein detection by dot blot

Exosomes and cell lysates were spotted on a nitrocellulose membrane (Thermo Fisher Scientific, UK) (0.5 µg protein in 40 µL for all samples – 10 µL at a time, dried under a nitrogen stream before addition of the next 10 µL on the same spot). The membrane was blocked with 3% milk (w/v) prepared in TBS-T (TBS pH 7.6 containing 0.1% Tween-20) for 1 h at RT. The membrane was then incubated with primary rabbit anti-human/mouse antibodies: anti-Alix (monoclonal, clone 3A9, ab117600, Abcam, UK), anti-TSG101 (polyclonal, 14497-1-AP, ProteinTech, UK), anti-CANX (polyclonal, 10427-2-AP, ProteinTech, UK) and anti-GAPDH (monoclonal, clone 14C10, #2118, Cell Signalling Technology, UK) antibodies (1:1000 in 3% milk), overnight at 4°C. The membrane was washed three times with TBS-T, followed by incubation with goat anti-rabbit secondary antibody (polyclonal, ab6721, 1:1000 in 3% milk) for 1 h at RT. The membrane was then washed again as above, and SuperSignal™ West Femto Maximum Sensitivity ECL substrate (Thermo Fisher Scientific, UK) was added to the membrane (50 µL per sample spot). The membrane was incubated with the substrate for 2 min at RT, and then imaged using the Gel Doc™ system (Bio-Rad, USA) under the “Intense Bands” setting. The image obtained was analysed using the Image Lab™ software (Bio-Rad, USA).

#### Morphology characterisation of exosomes

Freshly isolated exosome particles were used for electron microscopy observation. Scanning electron microscopy (SEM) was performed using FEI Inspect-F (Philips, Eindhoven, the Netherlands) equipment operated at 20 kV. Diluted exosome aliquots were fixed in 5% glutaraldehyde (Sigma-Aldrich) for 2 h and then incubated on the surface of (3-Aminopropyl)triethoxysilane(APTES) (Sigma-Aldrich) pre-treated silicon wafer for 1 h. The sample was then washed with PBS for three times and dehydrated in a series of increasing ethanol concentrations (20, 50, 70, 90, 95, 100%). Samples were then transferred for critical drying (Samdri, Tousimis). The samples were sputter coated with gold before SEM scanning.

Transmission electron microscopy (TEM) was performed using Philips CM 12 (FEI Electron Optics, The Netherlands) equipped with Tungsten filament and a Veleta – 2 k × 2 k side-mounted TEM CCD Camera (Olympus, Japan). The accelerating voltage was 80 kV. The spot size was set at 2. Objective aperture was used with all samples. Diluted exosome aliquots were fixed in 2.5% formaldehyde/glutaraldehyde (Sigma-Aldrich) in 0.1 M sodium cacodylate (Sigma-Aldrich) buffer, pH 7.4 for 15 min. Samples were then placed on 300 mesh carbon-coated copper grids (Agar Scientific, UK) until air dry. The samples were negatively stained with Millipore-filtered aqueous uranyl acetate (Agar Scientific, 25% in methanol) for 4 min followed by two 50% methanol/H_2_O wash. After air drying, the samples were observed under TEM.

### *Exosome fluorescence labelling* via *copper-free click chemistry*

Exosome suspension (200 µL, 2.8 × 10^12^ particle/mL) was mixed with 1 µL of 10 mg/mL of dibenzylcyclooctyne-NHS ester (DBCO-NHS, Lumiprobe) for 1 h in the dark at RT. Alexa Fluor^TM^ 488 azide (Life Technologies) (AF488-azide, 3.8 µL, 5 mg/mL) was then added and incubated for 4 h in the dark at RT. The molar ratio of exosome/DBCO-NHS/AF488-azide was 1:400:400. Unconjugated dye was removed by ultrafiltration using Nanosep® 300 K (Pall Life Sciences) or gel filtration using Sepharose CL-2B columns. For ultrafiltration, concentrated exosomes in the upper compartment of the Nanosep®column were resuspended with 200 µL PBS and collected in 1.5 mL microcentrifuge tubes after three consecutive centrifugations at 14,000 × g for 5 min at 4°C. For gel filtration, Sepharose® CL-2B (Sigma-Aldrich) as the resolving matrix was self-packed according to the dimensions of the commercially available NAP-5™ columns (Thermo Fisher Scientific) and optimised such that exosomes will elute in the first 2 × 500 µL fractions (F1 and F2). Then, 100 µL of labelled exosome solution was added to the column, followed by elution with 400 µL PBS (Fraction 0) and a series of elution with 500 µL PBS per fraction. Fractions 1 and 2 containing labelled exosomes were collected and stored at 4°C as a stock solution. Fluorescence intensity (FI) of labelled exosome (100 µL) was measured in black-walled 96-well plates using a FLUOstar Omega plate reader (Ex/Em: 485/520 nm) (BMG Labtechnologies GmbH, Germany). FI per exosome particle was calculated using the following formula:

$$\begin{array}{rl} & FI\;per\;exosome=\frac{FI\;per\;100\;\mu L}{100}\\ & \;\times \frac{\begin{array}{c} Volume\;of\;labelled\;exosome\;solution\;(\mu L) \end{array}}{\begin{array}{c} (Concentration\;of\;exosomes\times Volume\;used\;for\\ \;labelling\;(mL)\times Labelling\;efficiency\;(\%)) \end{array}} \end{array}$$

For ultrafiltration, labelling efficiency was determined by measuring the number of resuspended labelled exosomes by NTA compared with the number of unlabelled exosome stock. For gel filtration, labelling efficiency was determined by comparing the number of exosomes in Fraction 1 and 2 (1000 µL total) with the number of unlabelled exosome stock in 100 µL. The labelling efficiency was used to calculate FI per exosome and the number of labelled exosomes in stock solution.

### Measurement of fluorescent cells using flow cytometry

Cells (30 K) were cultured in Costar® 24-well flat-bottom plates and were incubated with labelled exosomes according to the chosen incubation times. Untreated cells were defined as negative control. Following incubation, cells were rinsed with sterile PBS, detached by trypsinisation, and resuspended in sterile PBS in polystyrene round-bottom tubes (Corning^TM^). Collected cells were analysed by flow cytometer (BD FACSCalibur^TM^, USA) with a minimum of 5,000 events acquired per sample. MFI was recorded. Degree of uptake by recipient cells was expressed in two different ways:

- Number of exosomes taken up by recipient cells, or Taken-up particle number ($N_{1}$):

$$N_{1}=\frac{\left(MFI_{treated\;cells}-MFI_{untreated\;cells}\right)}{FI\;per\;exosome}$$

(ii) The percentage uptake (% Uptake):

$$\%Uptake=\frac{N_{1}}{N_{0}}\times 100$$

where $N_{0}$ is the initial dose of exosomes incubated with the recipient cells. The taken-up particle number *N*_1_ (number of particles taken up by recipient cells) was calculated by dividing Delta FI (the difference between FI of untreated cells and FI of treated cells) with FI per exosome. Uptake efficiency (%Uptake) was expressed as   thepercentage of taken up exosome particles from the initial dose of exosomes added to the recipient cells.

### Design of experiments

Based on preliminary screening of cellular uptake of three types of exosomes in four cell lines (B16-F10, PANC-1, HPAC, and HEK-293), four factors were selected for cellular uptake simulation. These were defined as two qualitative factors (exosome type, cell type), and two quantitative factors, i.e. (dose of exosomes, incubation time). Multi-factor Design of Experiments (D-optimal design) was established using MODDE 10.1 software (Umetrics, Sweden) to investigate the effects of individual or 2-factor interactions on the responses at different levels. HPAC and PANC-1 cells were chosen as representative PC cells, whereas HEK-293 and B16-F10 cells were representatives of human non-cancer cells and non-human cancer cells, respectively. Based on the matrix of experimental design, 38 tests were designed and conducted in triplicate including three of them as centre points. Data fitting and calculation of statistical parameters (*R*^2^, *Q*^2^ and reproducibility) were performed by multiple linear regression (MLR) method. The experimental design used in this study allowed fitting the data with quadratic interaction model. Two responses investigated are the Taken-up particle number, and %Uptake.

### Dose-and time-dependent cellular uptake assay

In the case of time-dependency studies, PANC-1 cells were seeded in 6-well plates (Corning B.V., Netherlands) at a density of 300,000 cells per well overnight. The three types of labelled exosomes were then added at a dose of 2.0 × 10^10^ particles per well and incubated with the cells for 1, 4, 12, and 24 h at 37°C. Cells were then washed with warm PBS, fixed with 4% paraformaldehyde (PFA, Sigma-Aldrich) in PBS for 15 min at RT, and rinsed with PBS for three times. The cells were resuspended in 100 µL of PBS. Cell images (≥2,000 cells; bright field, dark field and fluorescence images) were acquired with an Amnis ImageStreamX MARK II (ISX) flow cytometer (Merck Millipore, Seattle, WA); A 488 nm wavelength excitation laser, at a power of 100 mW was used for excitation of the FITC fluorophore. Untreated cells were imaged as control. All image analysis was done using the Ideas software environment (Merck Millipore, Seattle, WA).

For dose-dependency studies, PANC-1 cells were seeded in Costar® 24-well flat-bottom plates at a density of 30,000 cells per well one day in advance. Doses of 4 × 10^9^, 1.2 × 10^10^, 2.0 × 10^10^, 2.8 × 10^10^, 3.6 × 10^10^ labelled exosomes derived from B16-F10, PANC-1 and HEK-293 cells were added to cultured PANC-1 cells and incubated for 24 h. The experiments were performed in triplicate. Cells were collected after incubation and analysed by flow cytometry.

### Confocal microscopy experiment

Cells were seeded at a density of 30,000 per well and grown to confluency overnight on glass coverslips in 24-well tissue culture dishes (Corning B.V., The Netherlands). Labelled exosomes were then added at a dose of 2.0 × 10^10^ particles per well and incubated for 1, 4, 12 and 24 h at 37°C. Cells were then washed with warm PBS and fixed in 4% PFA in PBS for 15 min at RT. For F-actin staining, cells were permeabilised with 0.1% Triton X-100 in PBS for 10 min at 4°C. After washing with PBS for 2–3 times, the cells were stained with methanolic phallotoxin (Alexa Fluor® 568 phalloidin, 578/600, Thermo Fisher Scientific, UK) for 20 min at RT according to the supplier’s protocol, followed by 2–3 times PBS washing. For nuclear staining, cells were treated with DAPI (300 nM in PBS, Sigma-Aldrich) for 1–5 min protected from light, then rinsed three times with PBS. Coverslips were mounted with Vectashield anti-fade mounting medium (Vector Laboratories Ltd, USA) and sealed with nail polish. Images were obtained using a confocal microscope (A1 R Si MP Confocal, Nikon, Japan) with a 60× oil immersion objective (Nikon, Japan) and fluorescence signals were captured *via* three bandpass filters (492 nm SP, 525/50 nm and 575/25 nm appearing in blue, green and red, respectively). For acidic organelles staining, cells were treated with prewarmed 75 nM LysoTracker Red DND-99 (577/590 nm, Thermo Fisher Scientific, UK) in fresh medium for 45 min at 37°C. After washing with PBS for 2–3 times, cells were fixed and nuclear stained as described above.

### In vivo uptake of the fluorescent PANC-1 exosomes

All *in vivo* experiments were conducted under the authority of project and personal licences granted by the UK Home Office and the UKCCCR Guidelines (1998). Male NOD SCID gamma (NSG) immunodeficient mice, 4–6 weeks old, were obtained from Charles River (UK). NSG mice bearing subcutaneous human pancreatic cancer (PANC-1) or mouse melanoma (B16-F10) were prepared to assess the tumour uptake of Cy7.5-labelled exosomes. Briefly, mice were anesthetised by isoflurane inhalation and injected subcutaneously with 5 × 10^6^ PANC-1 cells (100 μL in PBS) or 1 × 10^6^ B16-F10 cells (100 μL in PBS) at lower flanks using a sterile syringe with a 26-gauge needle. When tumours reached desired size (roughly 10 days after tumour implantation), mice were anaesthetized and injected intravenously (i.v.) with Cy7.5-Exo (8 × 10^11^ particles in 200 μL PBS) *via* a tail vein. At 24 h post-injection, the animals were perfused with 25 mL heparinised saline (1000 U/L) through the left ventricle of the heart to wash out residual or loosely bound exosomes in the circulation. All the major organs including liver, spleen, kidneys, heart, lungs, brain, stomach and intestine were then harvested. Excised organs were weighed, and their FI was measured using an IVIS Lumina Series III In Vivo Imaging System (Perkin-Elmer, UK). Fluorescence signals of the images were quantitatively analysed by drawing regions of interest around the tissues using the Living Image 4.3.1 Service Pack 2 software (Perkin-Elmer, USA). The results are expressed as total flux per organ (photons/s) or total photons per gram tissue (photons/s/g, mean ± SD).

### Statistical analysis

Quantitative results were presented as mean ± SD. Student’s unpaired t-test was utilised to compare control samples against experimental samples, using the geometric mean of each separate experiment. For multiple treated groups, statistical significance was examined using one-way ANOVA. Significance was specified as *p < 0.05, **p < 0.01, or ***p < 0.001.

## Results

### Exosome characterisation

**Figure 1.**
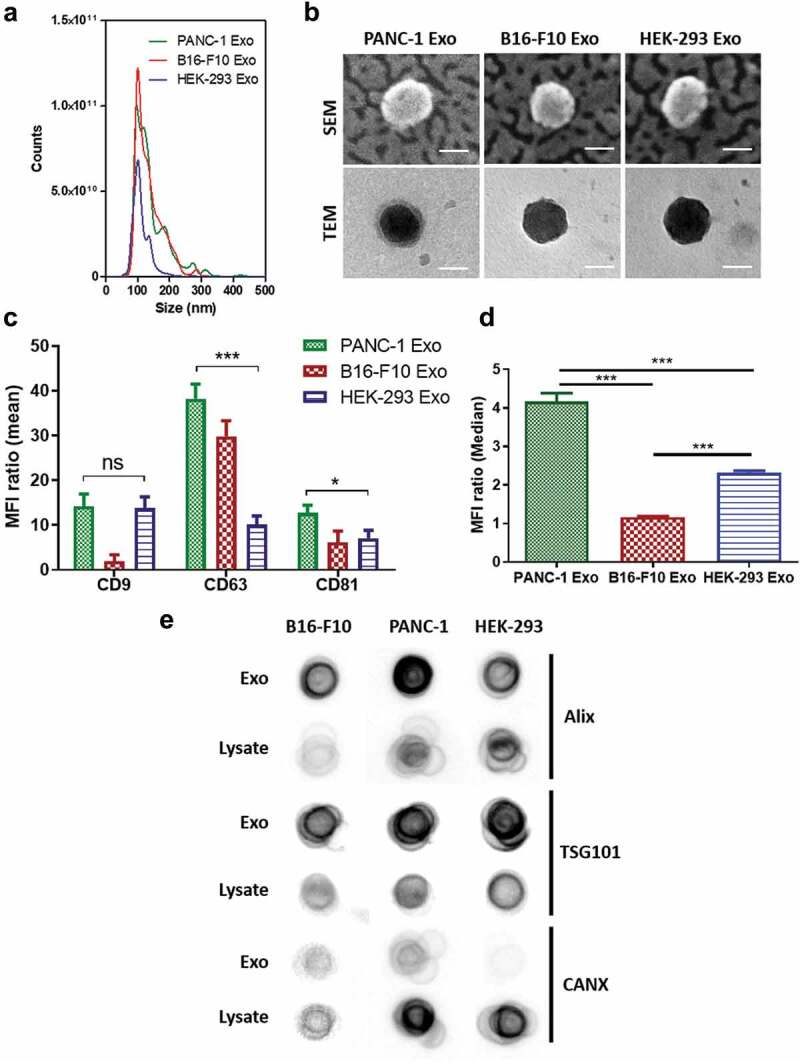
Characterisation of PANC-1, B16-F10 and HEK-293 Exosomes (Exo). (a) Size distributions of PANC-1, B16-F10 and HEK-293 Exo obtained by Nanoparticle Tracking Analysis (NTA) using a NanoSight LM-10. The histograms indicate a similar size distribution profile for all the three exosomes. (b) Morphology characterisation by transmission electron microscopy (TEM) and scanning electron microscopy (SEM). Scale bar: 50 nm. (c) Detection of tetraspanins CD9, CD63 and CD81 on exosomes using flow cytometry. Exosomes were coupled to aldehyde/sulphate latex beads prior to detection. Exo-beads complex were subsequently stained using a 2-step labelling (anti-CD9, anti-CD63 or anti-CD81 1° Ab/Cy5-conjugated 2° Ab). Degree of expression of the markers are expressed as the fold increase in mean fluorescence intensity (MFI) values to Exo-beads complex stained with Cy5-conjugated 2° Ab, where an MFI ratio of 2 was set as the threshold for positive expression. (d) Detection of “do-not-eat-me” marker CD47 on PANC-1, B16-F10 and HEK-293 Exo. A MFI ratio of 2 was set as the threshold for positive expression. Values are expressed as mean ± SD (*n* = 3). Statistical analysis were only conducted on human exosomes. *p < 0.05, ***p < 0.001, ns: No significance. (e) Dot blots for detection of luminal exosomal markers. Equal amounts of protein (0.5 μg in 40 μL) from the Exo and cell lysate samples for each cell line were spotted on nitrocellulose membranes. The membranes were then blocked with 3% milk, and stained using a 2-step labelling (anti-Alix, anti-TSG101 or anti-CANX 1° Ab/HRP-conjugated 2° Ab).

Physicochemical properties of exosomes isolated from PANC-1, B16-F10 and HEK-293 cells are summarised in Table 1. Size measurements using NTA showed exosomes from all cell lines are of ~100 nm in size, which fall in the expected exosome size range (30–150 nm) [3,11,25] (Figure 1(a)). There are no significant differences (ns) in size between the three types of exosomes. TEM and SEM analysis of the exosome samples revealed the presence of intact vesicular structures with lipid-bilayer membranes of similar sizes to that measured by NTA (Figure 1(b) & Figure S1) [26]. NTA measurements of exosome yield showed that exosomes from B16-F10 cell have the highest yield (1.0 ± 0.3 × 10^13^ particle/mL), followed by exosomes from PANC-1 cell (7.3 ± 0.3 × 10^12^ particle/mL) and HEK-293 cell (7.0 ± 0.2 × 10^12^ particle/mL). Zeta potential values recorded by PANC-1, B16-F10 and HEK-293 exosomes were −15.0 ± 1.8, −16.0 ± 2.6 and −16.7 ± 1.0 mV, respectively, which were not significantly different and comparable with other studies [25]. Protein concentrations in PANC-1, B16-F10 and HEK-293 exosome samples were 100.7 ± 9.3, 116.8 ± 9.1 and 84.3 ± 9.8 µg/mL, respectively. Particle:protein (P:P) ratio was used to assess the purity of the exosome samples from contaminating proteins co-precipitating from conditioned media during isolation. For all preparations, P:P ratio of > 2 × 10^10^ particle/μg protein was achieved, suggesting high purity of the exosome samples [27,28]. The expression of canonical tetraspanins CD9, CD81 and CD63 was confirmed on all exosome samples by flow cytometry (Figure 1(c)). The expression of the “do-not-eat-me” marker CD47 was also assessed on all exosome samples by flow cytometry. Both PANC-1 and HEK-293 Exo showed positive CD47 expression, with the former showing a significantly higher expression levels. CD47, however, was not expressed, if not only negligible, on B16-F10 Exo (Figure 1(d), Figure S2(a–c)). All exosome samples were found to be enriched for the expression of endosomal sorting complexes required for transport (ESCRT)-related proteins Alix and TSG101 as compared to the lysates of their cell of origin. Though Calnexin (CANX-an endoplasmic reticulum-associated protein) was slightly positive for exosomes from the B16-F10 and PANC-1 lines, it was much less enriched for each exosome compared to their cell lysate. As outlined in the MISEV2018 guidelines [29], this result could further confirm their endosomal origin and suggest minimal contamination of non-exosomal vesicles in the samples (Figure 1(e)). All exosome samples were also enriched for the expression of the cytosolic protein GAPDH, which is expected for certain cytosolic enzymes, and indicates the isolated exosomes as lipid bilayer structures with cytoplasmic material enclosed [29] (Figure S2(d,e)). In summary, exosome samples prepared in this study are of high yield and purity with regard to contaminating proteins from culture media and non-exosomal vesicles.

**Table 1.** Physicochemical characterisation of exosomes.

Exosome	Concentration (particle/mL) ^a,d,e^	Size (mode, nm) ^a,d,e^	Zeta potential (mV)^b,d,e^	Total protein concentration (µg/mL)^c,d^	Exosome to protein ratio (particles/µg) ^a,c^
PANC-1	7.3 ± 0.3 × 10^12^	101.0 ± 5.4	−15.0 ± 1.8	100.7 ± 9.3	7.3 × 10^10^
B16-F10	1.0 ± 0.3 × 10^13 **^	101.0 ± 2.0	−16.0 ± 2.6	116.8 ± 9.1	8.9 × 10^10^
HEK-293	7.0 ± 0.2 × 10^12^	107.0 ± 8.2	−16.7 ± 1.0	84.3 ± 9.8	8.3 × 10^10^

^a^Values were obtained using nanoparticle tracking analysis (NTA). Exosomes were isolated from 15 mL of supernatants from CELLine AD1000 bioreactor flasks. Pellets were resuspended in 0.4 mL PBS prior to NTA measurement.

^b^Samples were diluted 10 times with water before measurement by Zetasizer Nano ZS at 25°C.

^c^Quantified using micro BCA protein assay.

^d^Results are expressed as mean ± SD, where *n* = 3.

^e^One-way ANOVA was used for statistical analysis (ns: *p* > 0.05, p* < 0.05, p** < 0.01).

### *Fluorescence labelling of exosomes* via *click chemistry*

**Figure 2.**
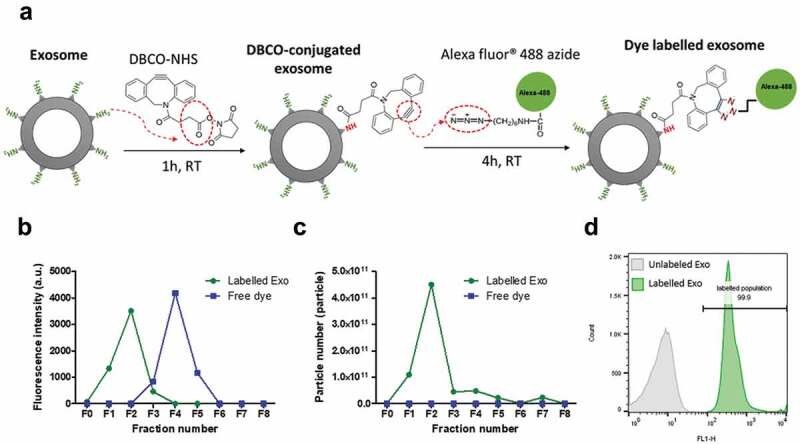
Clickable surface fluorescence labelling of exosomes and their characterisation. (a) Scheme of the fluorescence labelling method of exosome surface using copper-free click chemistry with AlexaFlour®488 (AF488)-azide. (b) Elution profiles (fractions 0–8 (F0–F8)) of labelled Exo or free dye using gel filtration with CL-2B column for purification. (c) NTA analysis of all the fractions from elution of both free dye and labelled Exo. Labelled Exo are collected from F1–F2. (d) Labelling confirmation by flow cytometry analysis of labelled Exo. Labelled and unlabelled Exo were conjugated to latex microbeads prior to analysis under the FL1 channel for detection of AF488 signals.

Fluorescence labelling was performed using a two-step labelling reaction (Figure 2(a)). The primary amine groups of lysine residues on exosomal surface proteins were firstly reacted with N-hydroxysuccinimidyl (NHS) group of DBCO in an aqueous environment. The alkyne group of DBCO was then coupled to the azide-functionalised AlexaFluor®488 (AF488) dye by copper-free click reaction. Three labelling protocols were compared (Figure S3), and the highest labelling yield was obtained when exosomes were first conjugated with DBCO-NHS for 1 h at RT and then immediately reacted with AF488-azide for 4 h at RT. This labelling protocol was used throughout the study.

To ensure complete removal of the unconjugated dye, size-exclusion chromatography (Sepharose® CL-2B column) and centrifugal ultrafiltration (Nanosep® 300 K) were compared for post-labelling purification (Table 2, Figure S4). The former resulted in ~8 times higher exosomal recovery (~78%), hence was chosen as the preferred purification method. Labelled exosomes eluted in the first 2 fractions as confirmed by spectrofluorimetry, NTA measurements and flow cytometry (Figure 2(b-d)). Higher FI was recorded at equimolar concentrations for B16-F10 exosomes (~4000 arbitrary unit per 100 µL of exosome dispersions) than PANC-1 and HEK-293 exosomes (~2000 arbitrary unit per 100 µL of exosome dispersions) (Figure S5). This translates into a higher dye per exosome molar ratios for B16-F10 exosome (~10) compared to that of PANC-1 and HEK-293 exosomes (~6), which potentially reflect the difference in availability of surface lysine groups on each exosome surface.

**Table 2.** Comparison of exosome purification methods post-labelling.

Exosome	Ultrafiltration (NanoSep)	Gel filtration (CL-2B column)	Labelling efficiency (%)^b,c^	Dye molecule per exosome ^b,c^
	Exosome recovery (%)^a,c^			
PANC-1	13.4 ± 2.7	78.8 ± 1.5	5.3 ± 0.2	6.4 ± 1.2
B16-F10	11.4 ± 2.3	79.4 ± 1.6	9.8 ± 0.5	9.5 ± 0.8
HEK-293	9.3 ± 1.7	77.0 ± 1.5	5.2 ± 0.1	6.1 ± 1.4

^a^Exosome recovery was calculated as the percentage of particles recovered from the original exosome number used. For ultrafiltration, the recovered particle number was obtained from NTA measurement of the retentate in the NanoSep filter column. For the gel filtration, the recovered number was obtained from NTA measurement of all fractions collected after elution.

^b^Calculations were based on gel filtration purification method. The labelling efficiency was calculated as the percentage of fluorescence signals in Fraction 1 and 2 (1,000 µL total) of total fluorescence intensity of exosome stock (100 µL) before purification. The labelling efficiency was used to calculate AF488 per Exo molar ratio.

^c^All measurements were carried out in triplicate and values are shown as mean ± SD.

Next, the stability of the labelled exosomes was assessed. The FIremained unchanged at 4°C storage for up to 3 months (Figure S5(a–b)). No significant differences in size, FI and zeta potential between naïve, freshly labelled or 3-month stored exosomes were observed (Figure S5, Table S1). Overall, this suggests that the copper-free click chemistry-based reaction allows fluorescent labelling of exosomes without altering their physicochemical stability and is, therefore, suitable for use in tracking the exosomes *in vitro* and *in vivo*.

### Design of experiment-driven experimental design and predictive model

**Figure 3.**
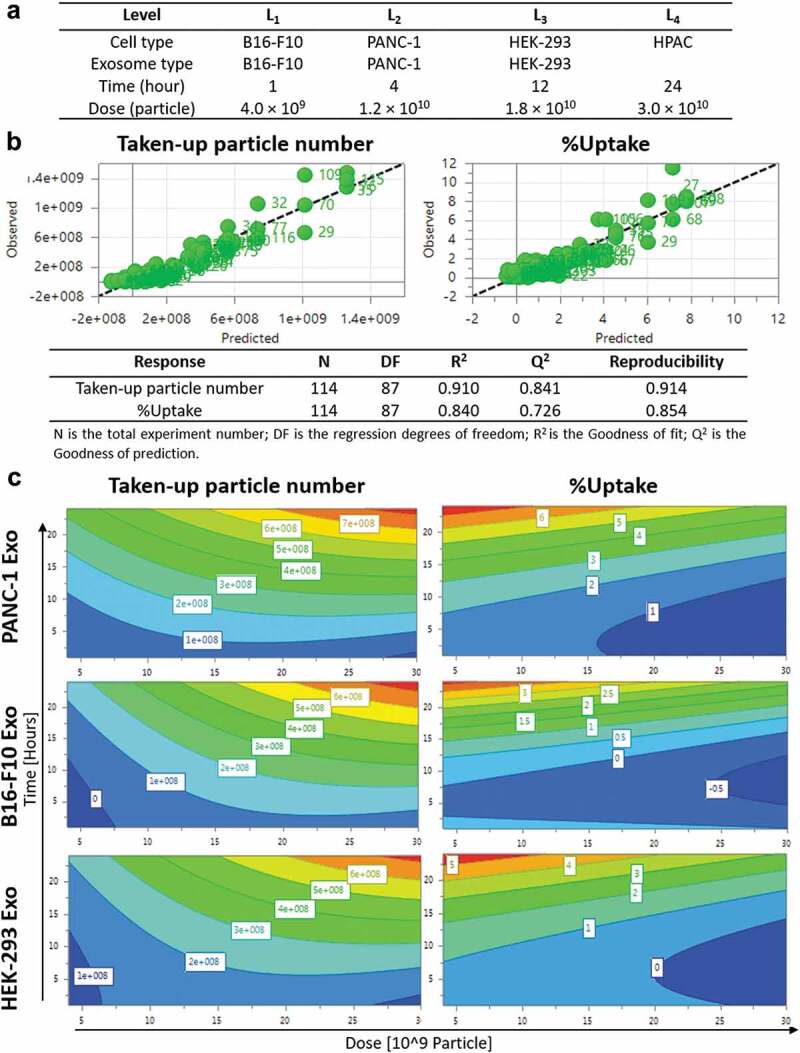
Design of experiments (DoE)-driven modelling of exosome cellular uptake. D-optimal design was established to combine four varied factors (exosome type, cell type, time and dose) at different levels to investigate the effects of individual factor or 2-factor interactions on the exosome cellular uptake. (a) Input information for establishing D-optimal Design using MODDE software. (b) Assessing the prediction power of the model in a plot of predicted vs. observed (with 1–1 line in black dashed line). Statistical parameters of the DoE model for Taken-up particle number and %Uptake modelling are summarised below the fitted data. (c) Contour plots relating the effect of incubation time and Exo dose on Taken-up particle number (left) and %Uptake (right) for PANC-1, B16-F10, HEK-293 Exo in PANC-1 cells. The red and darkest blue colour indicate the highest and lowest responses, respectively.

Design of Experiments (DoE) is an experimental design tool for optimisation studies and prediction of factor–factor interactions in a multiple-variable study based on mathematical modelling. Exosome uptake in cells is a complex phenomenon which involves multiple factors, and that the interaction between the different factors might also contribute differently to the uptake properties. Hence, the use of a powerful tool like DoE can be employed for the optimisation of such extensive experiments. The results from DoE modelling can also be used to predict the significance of the factor(s) studied. In this study, four recipient cell lines (PANC-1 and HPAC – human pancreatic carcinoma; B16-F10- murine melanoma; HEK-293- human non-cancer), three exosome types (PANC-1, B16-F10, HEK-293), four incubation time points (1, 4, 12, 24 h) and four exosome doses (0.4, 1.2, 1.8, 3.0 × 10^10^ particles) were selected as the four “variables” in a D-optimal DoE study to determine the extent of each effect independently as well as their interactions in influencing intracellular uptake of exosomes [30] (Figure 3(a)). Responses in this prediction model were the “Taken-up particle number” and “%Uptake”. Based on the DoE design, a total of 38 runs instead of 114 experiments were performed in random order using AF488-labelled exosomes obtained from the previous section and the results were uploaded back into the software to establish the predictive model. MLR regression analysis showed a good prediction quality with both *R*^2^ (Goodness of fit) and *Q*^2^ (Goodness of prediction) values of > 0.5, and the absolute difference between these two is <0.2 [30] (Figure 3(b)). The predictive model showed that both incubation time and exosome dose as important factors governing uptake, given by the highly positive coefficients for Taken up particle number (Figure S6, Table S2). The predictive model also showed that exosome-parent cell combination as an important factor for uptake of exosomes by pancreatic carcinoma cell lines, given by the positive coefficients recorded by PANC-1 and HPAC cells when paired with PANC-1 exosomes, but not when both cell lines were paired with exosomes derived from other cell sources (Figure S6, Table S2). A similar pattern was observed from the predictive model for B16-F10 cells - B16-F10 Exo pair indicating similar importance of this variable in influencing uptake. For HEK-293 cells, positive coefficients were observed when paired with both HEK-293 and PANC-1 Exo, suggesting a lower significance of exosome source in influencing cellular uptake. As this study aims to focus on determining important factors governing exosome uptake by PC cells, PANC-1 cell line was chosen as the model PC recipient for more in-depth subsequent analysis.

Contour plots obtained from the predictive DoE model showed both incubation time and exosome dose have a positive effect on the Taken-up particle number response for all exosome types in PANC-1 cells (Figure 3(c), left panel). The parameters in the white boxes of contour plots are the predicted Taken-up particle number at a certain time and dose, as generated by the software after modelling. This suggests that PANC-1 cells take up more exosomes regardless of their origin with increasing time or dose. The contour plots also indicate that the resultant effect of time and dose becomes more significant (red banding) at longer time point and higher dose range. Exosome source, however, showed a different effect to this measure of response. PANC-1 cells could take up more than 8 × 10^8^ PANC-1 Exo at the highest dose (3 × 10^10^) at 24 h. This was higher than that of either B16-F10 or HEK-293 Exo, both of which are predicted to be 7 × 10^8^ at highest dose. This observation is similar for other incubation time and exosome dose tested, suggesting that uptake of PANC-1 Exo was favoured by PANC-1 cells over other exosome types at any given dose or incubation time.

On the other hand, the contour plots showed that %Uptake of exosomes in PANC-1 cells increased with incubation time but decreased with increasing exosome dose, regardless of exosome sources (Figure 3(c), right panel). The parameters in the white boxes of contour plots are the predicted %Uptake at a certain time and dose, as generated by the software after modelling. Higher %Uptake was obtained at the lower end of the dose range. This is due to the smaller change in taken-up number (numerator for %Uptake) than the starting amount of exosome particle number (denominator for %Uptake). Interestingly, by comparison at 24 h and 5 × 10^9^ particle dose, PANC-1 exosomes recorded 7% uptake in PANC-1 cells, which is higher than the other two exosomes which only accounted for 4–5% uptake, respectively. Again, DoE modelling data suggest that PANC-1 Exo has a higher extent of uptake in PANC-1 cells at a given time or dose than other types of exosomes used in the model.

### Dose- and time-dependent cellular uptake of exosomes

**Figure 4.**
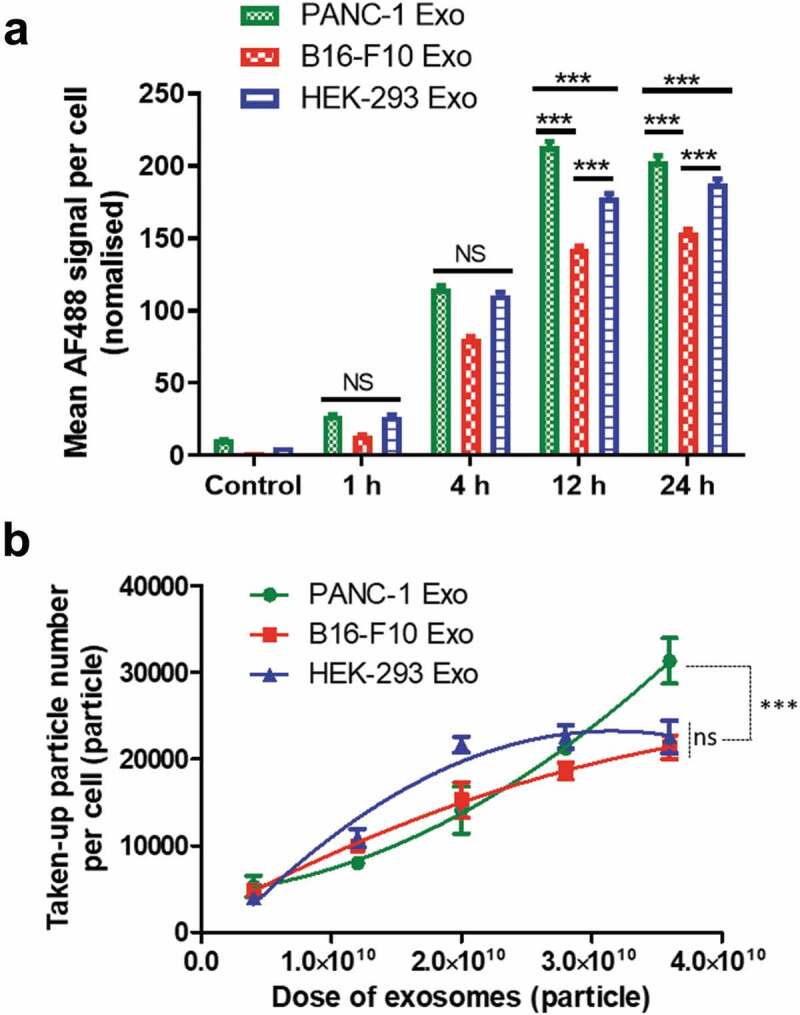
Effect of incubation time and exosome dose on *in vitro* exosome uptake in PANC-1 cells by imaging flow cytometry. (a) Time-dependent uptake profile of the three exosome types in PANC-1 cells. Each cell simultaneously imaged using the Imagestream Cytometer under the bright field and fluorescence (AF488) channels after incubation with labelled Exo for 1, 4, 12 and 24 h at a fixed dose of 2.0 × 10^10^ particles (sub-saturation level). Uptake was measured as the mean AF488 signal per cell, normalised according to the difference in labelling efficiency of the three exosomes. The error bars represent the s.e.m. (*n* > 2000 for all data). (b) Dose-dependent uptake profiles of the three exosome types in PANC-1 cells. PANC-1 cells were treated with each exosome at a dose of 4 × 10^9^, 1.2 × 10^10^, 2.0 × 10^10^, 2.8 × 10^10^, 3.6 × 10^10^ particles for 24 h. Uptake was measured as the taken-up particle number per cell. Values are presented as mean ± SD (*n* = 3). Statistical analysis was done for the two longest incubation times in (a), and the highest dose for (b).

Following the predictive modelling by DoE, the effects of single variables, namely, incubation time and exosome dose on the uptake of B16-F10, PANC-1 and HEK-293 exosomes in PANC-1cells were experimentally investigated. Effect of incubation time was studied first. All exosome types were fluorescently labelled with AF488 using the click chemistry-based approach, and incubated with PANC-1 cells at a dose of 2 × 10^10^ particles for 1, 4, 12and 24 h, respectively. Uptake in PC cells was quantified using imaging flow cytometry. Quantification of exosomes was based on image analysis in two different channels: bright field (BF, optical transmission) and fluorescence (AF488). Examples of the two image sets for four typical PANC-1 cells are shown in Figure S7. The morphology of fixed PANC-1 cells seen in the BF channel was different than that when they were adherent condition since they are now in suspension. Quantification of exosome uptake in PANC-1 cells was expressed as the MFI per cell in the AF488 channel. Values were normalised to account for initial differences in sample fluorescence intensities. Uptake of B16-F10 and HEK-293 Exoshowed a steady increase with incubation time from 1 to 24 h (Figure 4(a)). Uptake of PANC-1 exosomes also increased from 1 to 12 h but did not further increase at 24 h. At earlier time points such as 1 and 4 h, PANC-1 Exo showed no difference in uptake than that of HEK-293 Exo (p > 0.05), while at later time points (12 and 24 h), uptake of PANC-1 Exo was significantly higher than that of the latter (*p* < 0.001). All three exosome types showed no adverse effect on PANC-1 cell viability at 24 h post-incubation (Figure S8). This suggests that incubation time is a significant factor that influences exosome uptake by PANC-1 cells, and that the cells showed a preferential uptake of their “daughter” exosomes over other exosome types at longer incubation times e.g. 12 and 24 h.

Next, the effect of exosome dose was investigated. PANC-1 cells were incubated with each exosome type at a dose of 4 × 10^9^, 1.2 × 10^10^, 2.0 × 10^10^, 2.8 × 10^10^, 3.6 × 10^10^ particles for 24 h. The dose range was selected based on the DoE modelling data with an extension of the highest dose to 3.6 × 10^10^ particles, to study the effect of very high exosome doses on their uptake by PANC-1 cells. Their uptake was similarly assessed by imaging flow cytometry and was expressed as the Taken-up particle number per cell. Uptake of PANC-1 Exo and B16-F10 Exo showed a steady increase with increasing exosome dose, but that of HEK-293 Exo did not show further increase in uptake by PANC-1 cells beyond the dose of 2.8 × 10^10^ particles (Figure 4(b)). Uptake of PANC-1 was only significantly higher (*p* < 0.001) than the other two exosome types at the highest dose (3.6 × 10^10^ particles). This suggests that exosome dose is also an important factor governing exosome uptake by PANC-1 cells, and that the cells only showed a preferential uptake of their “daughter” exosomes at high doses. In summary, the imaging flow cytometry data validated the importance of incubation time and exosome dose in driving substantial exosome uptake by PANC-1 cells as predicted by DoE modelling, and that the selective uptake of PANC-1 Exo by their parent cell was only apparent at longer incubation time and higher exosome dose.

### Spatial distribution of exosomes internalised in PC cells

**Figure 5.**
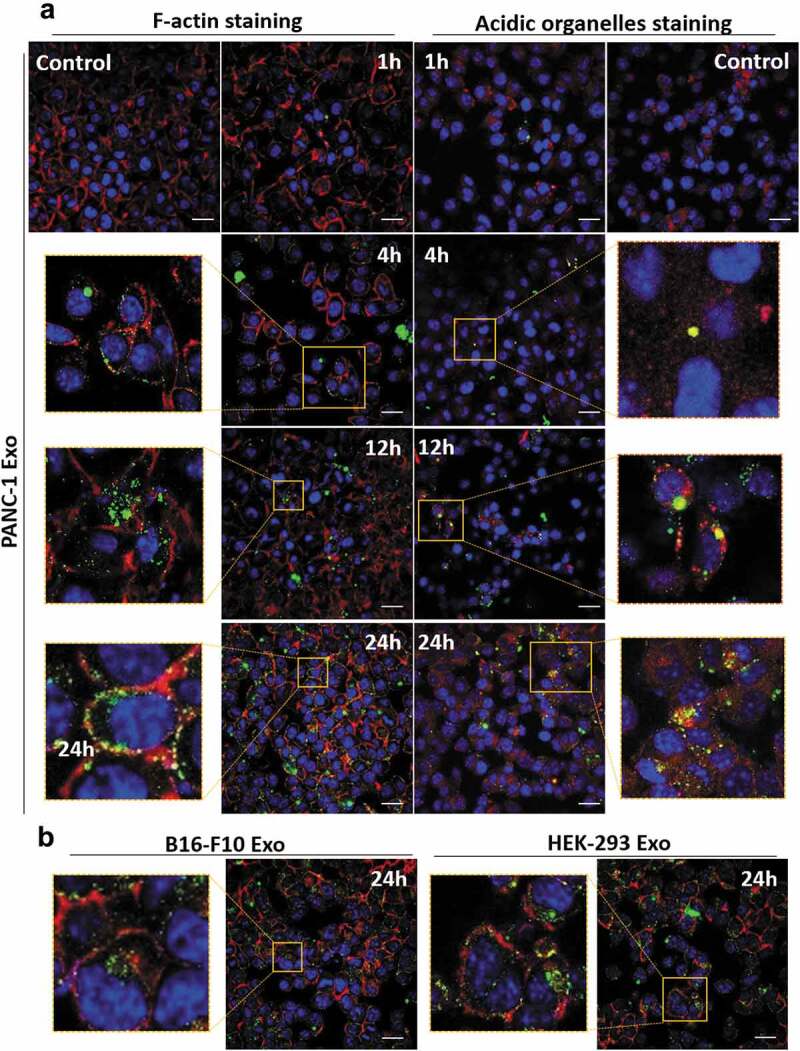
Spatial distribution analysis of Exo in PANC-1 cells by confocal laser scanning microscopy (CLSM). PANC-1 cells were seeded at 30 K per well in a 24-well plate overnight. Cells were then fixed, nuclei were counter-stained with DAPI (blue), F-actin were stained with AF568 phalloidin (red). (a) PANC-1 cells incubated with AF488-labelled PANC-1 Exo (green) at 2.0 × 10^10^ particles per well for 1, 4, 12 and 24 h at 37°C. In a separate experiment, acidic organelles (e.g. lysosomes, late endosomes) were stained with LysoTracker Red DND-99 (577/590 nm) (red). (b) PANC-1 cells incubated with B16-F10 Exo (left) or HEK-293 Exo (right) at 2.0 × 10^10^ particles per well for 24 h at 37°C. Scale bar: 20 µm.

Intracellular distribution of exosomes in PANC-1 cells at various time points (1, 4, 12 and 24 h) was studied using confocal laser scanning microscopy (CLSM). A median dose range of 2.0 × 10^10^ particles per well was used as the aim of CLSM study was to understand the intracellular distribution of exosomes, and not to compare the extent of their uptake. The nuclei were counter-stained with DAPI (blue), F-actin was stained with AF568 phalloidin (red) and the exosomes were labelled with AF488 (green) (Figure 5 and Figure S9). PANC-1 Exo were observed to be bound and internalised into the cytoplasm of PANC-1 cells in a time-dependent manner (Figure 5(a), left panel). To obtain more information on the spatial distribution of internalised exosomes, the acidic organelles of PANC-1 cells (e.g. lysosomes and late endosomes) were stained with LysoTracker Red (red) in a separate experiment. The yellow signals denote signal overlap i.e. accumulation of exosomes (green) in cellular acidic organelles (red), and were observed to be more pronounced at 24 h (Figure 5(a), right panel). Similarly using F-actin staining approach, B16-F10 and HEK-293 Exo also showed time-dependent uptake (Figure 5b and Figure S9). These data indicate that all three exosomes types were internalised into the cells and that this click chemistry-based surface labelling approach employed is reliable for *in vitro* tracking of exosomes.

### Substantial accumulation of PC-derived exosomes in pancreatic tumour xenografts after intravenous administration

**Figure 6.**
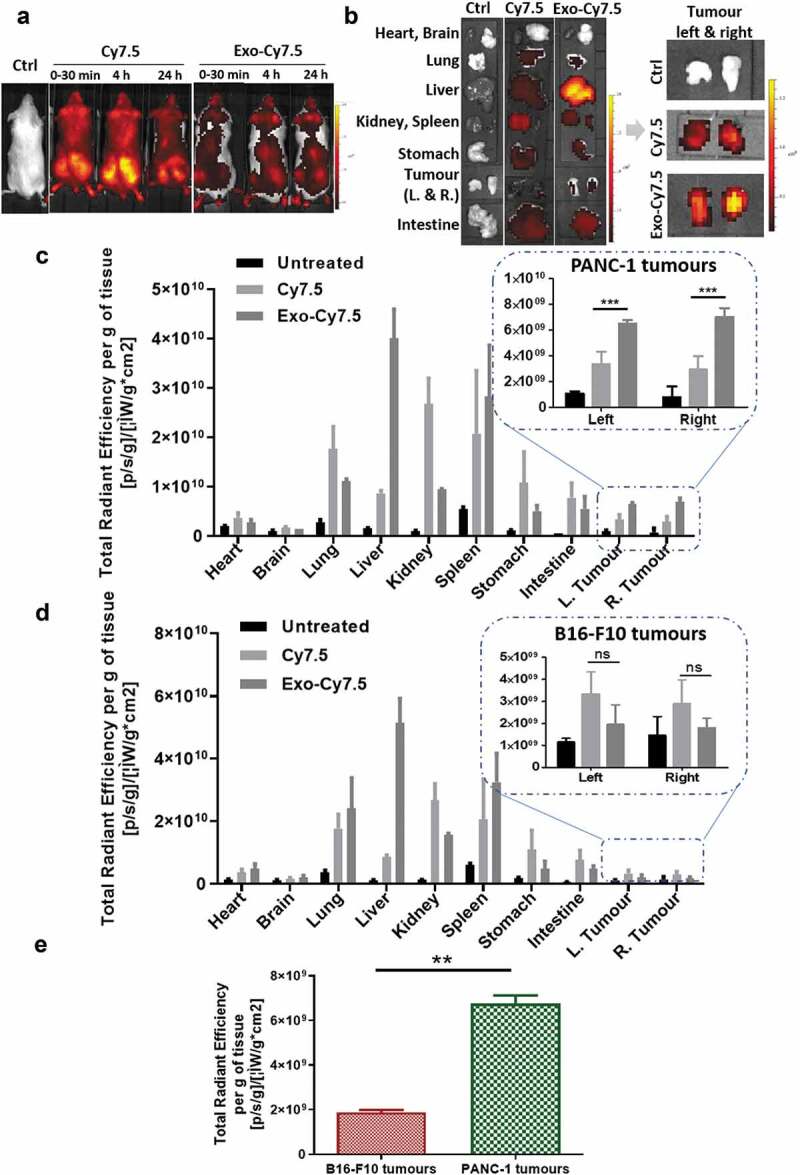
*In vivo* organ biodistribution profile of PANC-1 Exo in tumour-bearing NSG mice. Mice were inoculated subcutaneously with PANC-1 cells or B16–F10 cells in bilateral flanks (two tumours per mouse). Animals were intravenously injected with 200 µL containing PANC-1 Exo-Cy7.5 (approximately 8 × 10^11^ particles and 8.3 pmol of dye), Cy7.5 (approximately 8.3 pmol) or saline. Animals were killed at 24 h post-injection and the organs were excised for analysis. (a) Whole body live imaging, (b) *ex vivo* imaging and (c) organ biodistribution profiles of PANC-1 Exo in PANC-1 tumour-bearing mice. (d) Organ biodistribution profiles of PANC-1 Exo in B16–F10 tumour-bearing mice. For (c) and (d), Inset: zoomed-in tumour accumulation profile of PANC-1 Exo. (e) Comparison of PANC-1 Exo accumulation in PANC-1 vs B16–F10 tumours. Values were normalised to organ weight and expressed as mean ± SD (*n* = 3). Statistical analysis was done on tumour accumulation values. (ns: no significance, **p < 0.01, ***p < 0.001).

Following encouraging *in vitro* results suggesting the potential favoured uptake of PANC-1 Exo by their parent cells, uptake studies of PANC-1 Exo by self-tissue were carried out in PANC-1 tumour-bearing mice to determine if such preferential uptake is still apparent *in vivo* when a high dose is administered after 24 h. PANC-1 Exo was labelled with Cy7.5 dye using the in-house click chemistry-based labelling and administered intravenously into the mice (8 × 10^11^ particles/animal). Whole body imaging carried out at 30 min, 4 h and 24 h post-injection showed a significantly different biodistribution pattern between the free Cy7.5 dye (control) and Cy7.5-labelled PANC-1 Exo, confirming successful and stable exosome labelling (Figure 6(a)). This was supported by the *ex vivo* imaging and normalised tissue fluorescence quantification at 24 h where significantly higher liver accumulation was observed in mice administered with labelled Exo as compared to that with free dye, which is expected for spherical nanoparticles in the range of 100–200 nm in size (Figure 6(b,c)). The semi-quantitative biodistribution analysis showed that PANC-1 Exo recorded the highest accumulation in the liver and spleen, followed by the lungs and kidneys at 24 h post-injection, which was comparable to other studies (Figure 6(c)). Interestingly, substantial accumulation of PANC-1 Exo was observed in self-tissue (i.e. PANC-1 tumour mass), and was significantly higher than the signals obtained in the tumours of mice administered with free dye, again confirming that fluorescence observed in the tumours was indeed from labelled PANC-1 Exo. To discern whether uptake of PANC-1 Exo in PANC-1 tumours *in vivo* is enhanced permeability and retention (EPR)-dependent or self-tissue tropism, Cy7.5-labelled Exo was intravenously administered (also 8 × 10^11^ particles/animal) in mice bearing the more vascularised B16-F10 subcutaneous tumours. In this tumour model, PANC-1 Exo showed a similar biodistribution in general with predominant accumulation in the liver, spleen, lungs and kidneys (Figure 6(d)). Whole body and *ex vivo* imaging of data for PANC-1 Exo in B16-F10-bearing mice are shown in Figure S10. However, PANC-1 Exo showed a much lower accumulation in B16-F10 tumours, and that the values were significantly lower than that in PANC-1 tumours despite the former being more vascularised (Figure 6(e)). In summary, PANC-1 Exo showed substantial accumulation in their self-tissue *in vivo* when administered at a high dose at 24 h post-administration and that the accumulation was reliant on self-tissue tropism, which was consistent with the *in vitro* uptake studies. This also validated the reliability of the click chemistry labelling approach for *in vivo* exosome tracking and quantification.

## Discussion

In order to be used in a pre-clinical and clinical setting, a streamlined exosome isolation platform is highly needed [31]. In this study, ultracentrifugation onto a sucrose cushion was used to isolate exosomes from cell culture supernatant or CM, after prior removal of cell debris using differential centrifugations and larger vesicles by passing the CM through a 0.22 µm filter. This protocol can produce high-quality exosomes, as characterised and proved in the downstream applications such as labelling and uptake studies. Nevertheless, it is not suitable for isolating extracellular vesicles (EVs) from large volumes which are required in clinical settings [31]. A more practical approach, i.e. dealing with large volumes yet yield pure EVs is still required for isolating exosomes directly from CM, as well as from biological fluids in clinical settings.

In this study, presence of CD9, CD63 and CD81 on exosomes was confirmed by flow cytometry. Dot blot, however, was used for the verification of the presence or absence of luminal exosomal markers (Alix, TSG101, CANX, and GAPDH). The MISEV 2018 stated by the International Society for Extracellular Vesicles (ISEV) did not exclude dot-blot for protein analysis in EVs [29]. It is worth emphasising that Western Blotting is a more suitable method than dot blot to verify the exact molecular weight of the marker protein bands and will be advisable to use in subsequent studies instead of dot blot. In our study, we took care to block the nitrocellulose membranes using an optimised blocking buffer (3% milk) to minimise the non-specific binding from both primary and secondary antibodies.

As described earlier, fluorescence labelling of exosomes commonly done using lipophilic dyes is associated with various drawbacks that can lead to major result misinterpretations. The click-chemistry surface labelling approach proposed in this study is able to overcome such drawbacks, while still providing good labelling stability (*in vivo* and 3 months post-labelling at 4°C) and maintenance of exosomal integrity post-labelling (Figure S5) that can ensure reliable interpretation of subsequent results. It was also mentioned earlier that the unique array of transmembrane proteins expressed on exosomes play important roles in their intracellular uptake, and so the covalent attachment of the initial NHS-DBCO linker bears the risk of disrupting important binding epitopes of these exosomal proteins and therefore affecting cellular uptake of the exosomes *in vitro* and *in vivo*. Previous work using a similar approach for membrane radiolabelling of exosomes showed that the initial NHS-amine reaction resulted in only minimal disruption of exosomal surface protein epitopes, again ensuring the reliability in the uptake results obtained from studies using this labelling approach [32]. Although use of nuclear modality for exosome imaging and uptake quantification will offer more robust and accurate quantitative results due to limitations associated with the optical modality such as 2D acquisition of signals and variable tissue penetration depth of fluorescent signals [32], access to nuclear-based techniques and equipments is often limited in the majority of research labs. Hence, fluorescent-based imaging and quantification of exosome uptake *in vitro* and *in vivo* remain a relevant and more feasible technique. The surface exosome fluorescence labelling approach developed in this study provides a great tool to ensure comparability and reliability of results obtained from such studies.

Click chemistry has been previously reported for labelling both exosomes [33,34] and their parent cell lines [35]. For example, a facile metabolic incorporation of azide-labelled glycan on exosomes was developed, of which the azide-functionalised exosomes are then labelled using bio-orthogonal copper-free click chemistry for tracking. This approach requires prior modification of the parent cells (i.e. feeding the cells with tetra-acetylated N-azidoacetyl-D-mannosamine for the metabolic incorporation) from which the exosomes are derived from, and therefore will not be applicable to exosomes derived from biological fluids. In comparison, our proposed surface fluorescence labelling approach relies on covalent conjugation of the NHS-DBCO linker onto primary amines on lysine residues of exosomal transmembrane proteins. Lysine residues are found to be three times more abundant than cysteine residues in living organisms, and so are ideal sites for bioconjugation [36]. The surface labelling approach proposed in this study is, therefore, a universal method that can be applied for labelling any exosomes, regardless if they are sourced from cell cultures or biological fluids. The reaction between primary amines with active esters such as N-hydroxysuccinimide (NHS) used in this study or sulpho-NHS esters to form stable amide bonds, as well as the copper-free DBCO-azide click reaction occurs rapidly in aqueous solutions at physiological pH [34,36,37], thus making this labelling approach ideal in terms of only requiring simple and mild reactions without the risk of damaging the exosomes. The click chemistry-based fluorescence labelling of exosomes used in this study also showed about 10-fold higher labelling efficiency compared to directly conjugating AF488-NHS to exosomes in our previous work [32]. A short aliphatic carbon chain links the DBCO to the NHS groups in this bifunctional molecule, acting like a spacer between these two moieties. This spacer, together with the much smaller DBCO moiety potentially minimise steric hindrance for the NHS moiety on this molecule to access available amine groups which might be buried deeper within the extraluminal domains of exosomal transmembrane proteins, thereby increasing the rate of the DBCO conjugation to the exosome surface and therefore labelling efficiency as compared to that of the NHS moiety directly attached to a bulkier dye molecule e.g. AF488. A slight outward extension away of the DBCO moiety from exosome surface by the aliphatic chain spacer also potentially minimises similar steric hindrance issue in its subsequent reaction with the azide-functionalised bulky dye molecule, again contributing towards higher labelling efficiency. The primary step of DBCO conjugation to exosomal surface also offers versatility for this approach to be used for other applications such as radiolabelling or other exosome surface functionalisation by varying the azide-functionalised molecules reacted with the conjugated DBCO (e.g. azide-NOTA for radiolabelling).

The latest statement from ISEV community highlighted the need for a quantitative understanding of exosome uptake by cells [29]. Factors determining the degree of exosome uptake by recipient cells are yet to be properly outlined. To the best of our knowledge, this is the first study to use DoE for the prediction of single or multifactor interaction in parameters that potentially influence cellular uptake of exosomes. Use of DoE in this study enabled a more rational experimental design, where the number of relevant experiments required was reduced from 114 to 38 (3 exosomes, 4 cell lines, 4 time points and 4 doses). The DoE model predicted incubation time and exosome dose as significant factors affecting uptake of all exosome types used in this study, and that both factors can contribute synergistically towards exosome uptake. This was validated by the actual *in vitro* uptake studies, demonstrating the reliability of DoE modelling as a tool for designing multivariable optimisation studies. DoE did not generate an uptake profile (curve) for each of the investigated exosomes, but DoE did predict that PANC-1 Exo showed the highest uptake at the high doses. For example, PANC-1 cells could take up more than 8 × 10^8^ PANC-1 Exo at the highest dose (3 × 10^10^) at 24 h. This was higher than that of either B16-F10 or HEK-293 Exo, both of which were predicted to take up 7 × 10^8^ particles at the highest dose. Interestingly, the DoE model also predicted that exosome-parent cell pairing as an important factor in influencing exosome uptake a PC context. This was also validated in the actual *in vitro* uptake studies, but such favoured accumulation of PC-derived exosomes by their parent cells was only apparent at higher exosome dose and longer incubation time. It was reported that smaller exosomes are taken up more efficiently by cells [38]. No significant differences in size or charge were found between the three types of exosomes used in our study (*p* > 0.05) so it is unlikely that differences in uptake profiles are size- or charge-dependent. This suggests that PC cells show cell-specific preference towards internalising self-exosomes, which was also observed in other contexts such as in ovarian cancer models [19]. As indicated by *in vitro* exosome uptake study, the difference in uptake kinetics could be related to different uptake mechanisms. Many studies supported the hypothesis that exosomes are primarily taken up by receptor-mediated endocytosis [16,21,39,40]. We hypothesise that different exosomes were taken up by PC cells through different pathways. The main uptake pathway of HEK-293 Exo in PANC-1 cells could be receptor-mediated, as suggested by the saturable uptake profile of HEK-293 Exo potentially due to the limited availability of free surface receptors on PC cell membrane at high exosome dose, as receptor-mediated endocytosis requires specific interactions between the ligands and the receptors on the recipient cells. B16-F10 and PANC-1 Exo could have exploited multiple endocytosis pathways (e.g. clathrin or caveolae-mediated/independent endocytosis, or micropinocytosis) in PANC-1 cells as they do not show such saturable uptake. However, broader and more in-depth comparative studies are needed to conclude whether this preferential uptake holds for other PC cell lines, and that if the exosome-parent cell pairing is also important in selective uptake of tumour-derived exosomes in other cancer contexts. Specific protein(s) or lipid(s) component of the cell or exosomes that is important for the selective internalisation can then be determined to elucidate further understanding on the biochemical factors governing exosome uptake.

The doses used in the *in vitro* studies are in the range of 0.4–3.6 × 10^10^ particles for 30 K PC cells seeded in cell culture plate. For the *in vivo* study, it was assumed that there is a much higher number of cells (at least by an order of magnitude) in the implanted subcutaneous PC tumour than that from the 30 K cells used *in vitro*. Therefore, a higher dose of 8 × 10^11^ particles per animal was used to ensure a comparable amount of exosomes of EVs were administered. This single *in vivo* dose corresponds to approximately 11 µg (in terms of protein amount). It was reported that a higher dose of 60 μg of PC cells-derived exosomes had no observable side effects on Balb/c and NOD.CB17-Prkdc scid/J mice [41], and similarly the dose used in this work also did not give rise to any adverse effects in the mice.

The preferential uptake of PANC-1 Exo by their parent PANC-1 cells was recapitulated *in vivo*, as demonstrated by the significantly higher uptake of the former in PANC-1 tumours than that in the more vascularised B16-F10 tumours (Figure 6) [42]. It is worth noting that although B16-F10 cells were predicted by the DoE modelling to show preferential uptake of self-B16-F10 Exo *in vitro* in this study, which was observed with actual experimental investigation in our previous work, preferential uptake by B16-F10 tumours was not observed *in vivo* in the same previous study [32]. It was described earlier that expression of CD47 on exosome membranes confers protection from clearance by circulating macrophages and therefore prolongs the circulation time of exosomes *in vivo* [5]. CD47 expression was significantly higher on PANC-1 Exo than that on B16-F10 Exo (negligible expression) (Figure 1(d)). This suggests that exosome circulation time is an important enabling factor for manifestation of preferential uptake of exosomes by self-tissue *in vivo*. This finding also highlights the importance of assessing the expression of CD47 on exosomes as a selection criteria for suitability of a particular exosome type to be used as *in vivo* drug nanocarriers. The preferential uptake of unmodified self-exosomes by PC cells is therefore desirable for applications as nanocarriers for therapeutic delivery against PC, but warrants investigation into the risk of such strategy being counter-productive in terms of treatment efficacy due to the implication of tumour-derived exosomes in promoting tumour progression and metastasis [43]. Treatment efficacy in pre-clinical PC models using PC-derived exosomes as nanocarriers should also be correlated with the extent of tumour accumulation of the unmodified exosomes, to determine if endowment of active targeting ligands specific for certain surface proteins overexpressed in PC (e.g. αvβ6 integrin) on the exosomes are required [44]. The click-chemistry-based surface labelling approach proposed in this study would serve as an excellent tool for such studies.

## Conclusions

In this work, we have reported a novel yet simple, reliable and universal method to fluorescently label exosomes without prior engineering on parent cells for tracking and quantification of their intracellular uptake *in vitro* and *in vivo*. We have also demonstrated that DoE is a useful and reliable tool for a multivariable study design and prediction of important factors governing uptake of different exosome types by PC cells. This has enabled us to validate exosome dose and incubation time as important factors that can positively influence exosome uptake into PC cells regardless of their cell sources. We also reported the preferential *in vitro* uptake of self-exosomes in a PC context. This preferential uptake of self-exosomes by PC cells was also recapitulated *in vivo*, and that the expression of CD47 on the exosomes is crucial for the manifestation of such favoured uptake *in vivo*. Findings from this work would serve as the framework for future rational design of exosomes as nanocarriers for delivery of therapeutics against the different forms of PC and other cancer contexts.

## Supplementary Material

Supplemental Material

## Supplementary material

The supplemental data for this article can be accessed here.
