# Supplementary material for: Design of experiment (DoE)-driven in vitro and in vivo uptake studies of exosomes for pancreatic cancer delivery enabled by copper-free click chemistry-based labelling
Source: J Extracell Vesicles. 2020 Jun 19;9(1):1779458. doi: 10.1080/20013078.2020.1779458 (PMC7480572; doi:10.1080/20013078.2020.1779458)
Supplement: Supplemental Material [file ZJEV_A_1779458_SM2659.docx]

Supplementary Information

Design of experiment (DoE)-driven *in vitro* and *in vivo* uptake studies of exosomes for pancreatic cancer delivery enabled by copper-free click chemistry-based labelling

*Lizhou Xu^a^, Farid N. Faruqu^a^, Revadee Liam-Or^a^, Omar Abu Abed^a.b^, Danyang Li^a^, Kerrie Venner^c^, Rachel J Errington^d^, Huw Summers^e^, Julie Tzu-Wen Wang^a^, Khuloud T. Al-Jamal^a,^ **

^a^ School of Cancer & Pharmaceutical Sciences, Faculty of Life Sciences & Medicine, King's College London, Franklin-Wilkins Building, 150 Stamford Street, London SE1 9NH, United Kingdom

^b^ Health Science Department, Faculty of Graduate Studies, Arab American University in Palestine, Ramallah, Palestine

^c^ Institute of Neurology, University College London, Queen Square, London WC1N 3BG, United Kingdom

^d^ Division of Cancer and Genetics, School of Medicine, Cardiff University, Heath Park, Cardiff, CF14 4XN, United Kingdom

^e^ College of Engineering, Swansea University, Bay Campus Fabian Way, Crymlyn Burrows Swansea, SA1 8EN, United Kingdom

Corresponding author @:

Prof. Khuloud T. Al-Jamal

Email address: khuloud.al-jamal@kcl.ac.uk (K.T. Al-Jamal).

## **Supporting Methods**

## ***Zeta potential measurement***

The zeta potential of purified exosomes and labelled exosomes in PBS were measured using a Zetasizer Nano Series ZS with illumination from a 633 nm He-Ne laser (Malvern Instruments, Malvern, UK). Samples were diluted into approximately 2 × 10^8^ particles/mL concentration using 10 times diluted PBS and then transferred to a disposable plain folded capillary Zeta cell. Zeta potential was determined from the electrophoretic mobility using the Smoluchowski approximation. Measurements were carried out at room temperature.

## ***Cell viability test with MTT assay***

PANC-1 cells were seeded onto 96-well plates (1 × 10^4^ cells/well) 24 h prior treatment with labelled exosomes at a concentration of 2.0 × 10^10^ particles per 30K cells. Cells treated with 10% DMSO were used as a cytotoxic control for the MTT assay. At 24 h post incubation, MTT assay was performed. Cells were incubated with MTT solution at 0.5 mg/mL MTT final concentration for 3 h at 37°C and 5% CO_2_. Media was then removed, and the formazan produced was dissolved in 200 μL DMSO and absorbance was read in a FLUOstar Omega (BMG labtech, UK) plate reader at 560 nm. Results were expressed as the percentage of cell viability (mean ± SD) and normalized to naïve cells.

## **Supporting Results**

## ***Optimisation of click chemistry-based labelling methods***

Three different labelling methods were optimised, as described below and shown in **Figure S3A**.

**Method A**: Exosomes were first conjugated with DBCO-NHS at RT for 1 h (protected from light) at the NH_2_-NHS mole ratio of 1:400, and then reacted with AlexaFlour®488-azide by copper-free click chemistry at RT for 4 h (protected from light) at the mole ratio of alkyne-azide of 1:1.

**Method B**: Exosomes were simultaneously conjugated with DBCO-NHS and Alexa Fluor 488-azide at RT for 4 h (protected from light) at the same mole ratio of NH_2_-NHS and alkyne-azide as Method A.

**Method C**: After exosome-DBCO-NHS conjugation (same as Method A), the free DBCO-NHS was washed away by ultrafiltration, followed by the click chemistry reaction (same as Method A).

All the methods were followed by purification, either using ultrafiltration (Nanosep® 300K) or gel filtration (Sephalose® CL-2B column), which was optimised below.

In method C, after exosome-NH2/DBCO-NHS conjugation, the free/unlabelled DBCO-NHS were washed away by ultrafiltration, followed by the click chemistry reaction. In method A, this washing step was not included. Method A was deemed as an optimal method to use. One may think that method C would be a more efficient labelling method, however the unavoidable loss associated with the ultrafiltration step resulted in less amount of EVs available for the click reaction hence resulted in an overall less labelling yield.

## **Supporting Figures**

**
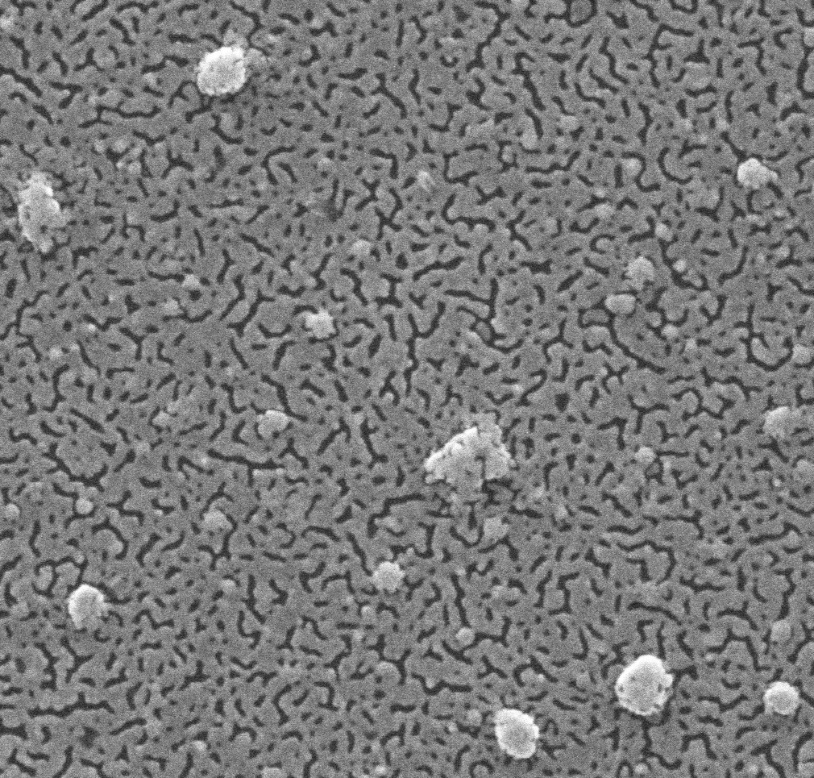
**

200 nm

**A**

**B**

**Figure S1. (A) Scanning electron microscopy (SEM) and (B) transmission electron microscopy (TEM) images of exosomes**. Scale bar: 200 nm.


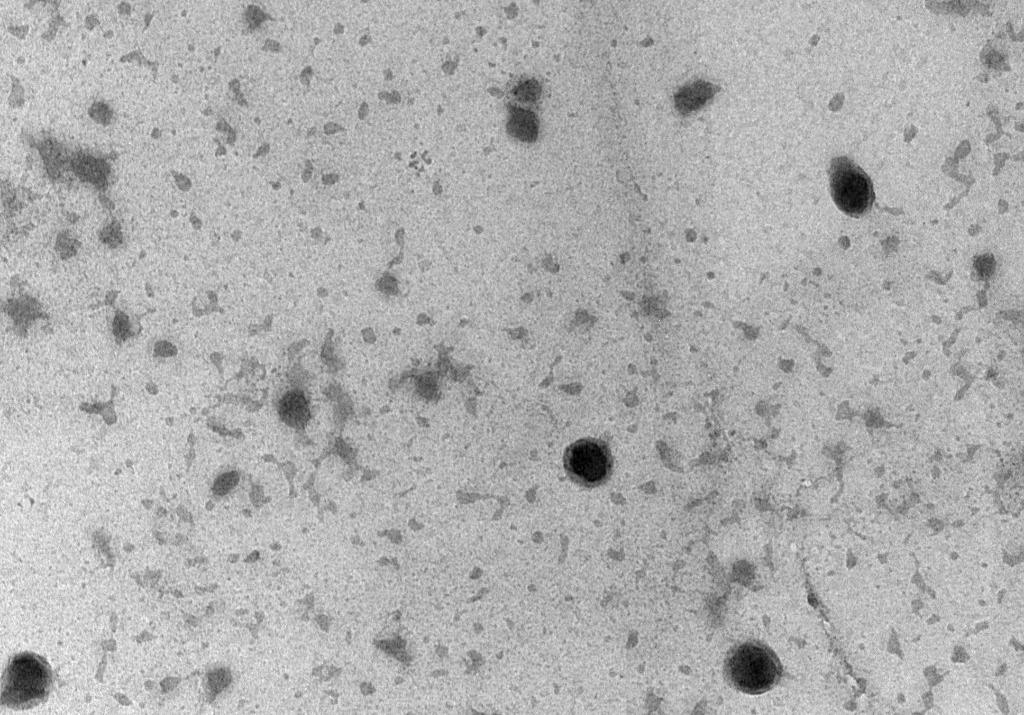


200 nm

**
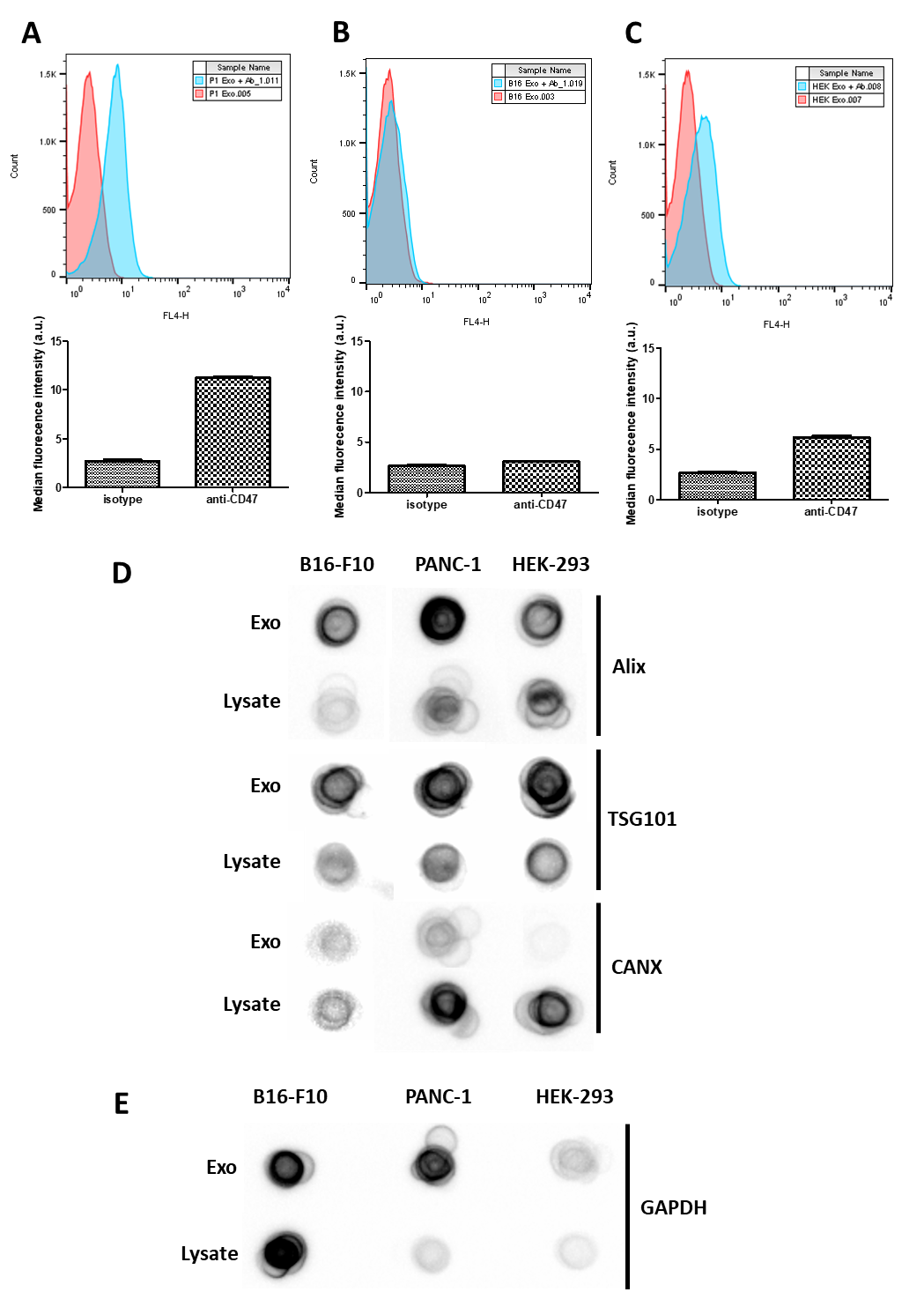
**

**Figure S2**. **Biochemical analysis of exosome samples. (A)**, **(B)** and **(C)** Detection of ‘don’t-eat-me’ marker CD47 on PANC-1, B16-F10 and HEK-293 Exo by flow cytometry, respectively. Exosomes were coupled to aldehyde/sulphate latex beads prior to detection. PANC-1 and HEK-293 Exo-beads complex were stained with APC anti-human CD47 antibody. APC anti-human isotype antibody was used as control. B16-F10 Exo-beads complex was stained with APC anti-mouse CD47 antibody. APC anti-mouse isotype antibody was used as control. Histograms indicate the shift in fluorescence signal, while the bar charts denote the numerical median fluorescence intensities of each Exo sample. Degree of expression of the markers are expressed as the fold increase in median fluorescence intensity (MFI) values from that of the isotype control (see **Figure 1D**). (D) Dot blots for detection of luminal exosomal markers. Equal amounts of protein (0.5 μg in 40 μL) from the Exo and cell lysate samples for each cell line were spotted on nitrocellulose membranes. The membrane was then blocked with 3% milk, and stained using a 2-step labelling (anti-Alix, anti-TSG101 or anti-CANX 1° Ab/HRP-conjugated 2° Ab). (E) Dot blots for detection of luminal GAPDH in PANC-1, B16-F10 and HEK-293 Exo. Equal amounts of protein (0.5 μg in 40 μL) from the Exo and cell lysate samples for each cell line were spotted on nitrocellulose membranes. The membranes were then blocked with 3% milk and stained using a 2-step labelling (anti-GAPDH 1° Ab/HRP-conjugated 2° Ab).

**
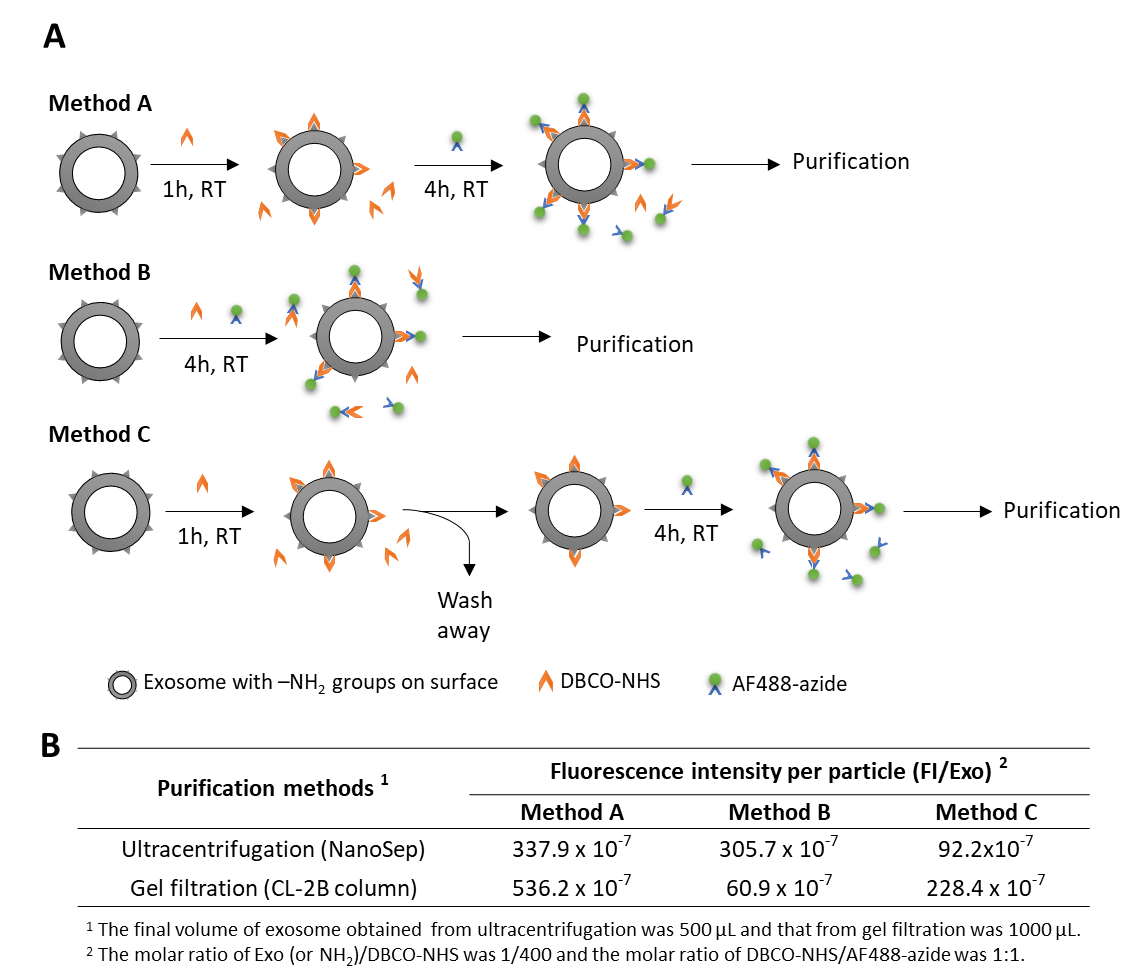
**

**Figure S3**. **Optimisation of the copper-free click chemistry labelling protocol and purification method.** (A) Schematic illustration of the three labelling methods compared. **Method A**: Exosomes were firstly conjugated to DBCO-NHS and then labelled with AF488-azide by click chemistry. **Method B**: Exosomes were simultaneously reacted with DBCO-NHS and AF488-azide. **Method C**: After Exo/DBCO-NHS conjugation, free DBCO-NHS was washed away, followed by click reaction of the AF488-azide. Labelled Exo was purified by ultracentrifugation using NanoSep® (100K) or gel filtration using Sepharose CL-2B columns for comparison. (B) A summary of fluorescence intensity per particle (FI/Exo), resulted from different labelling methods.


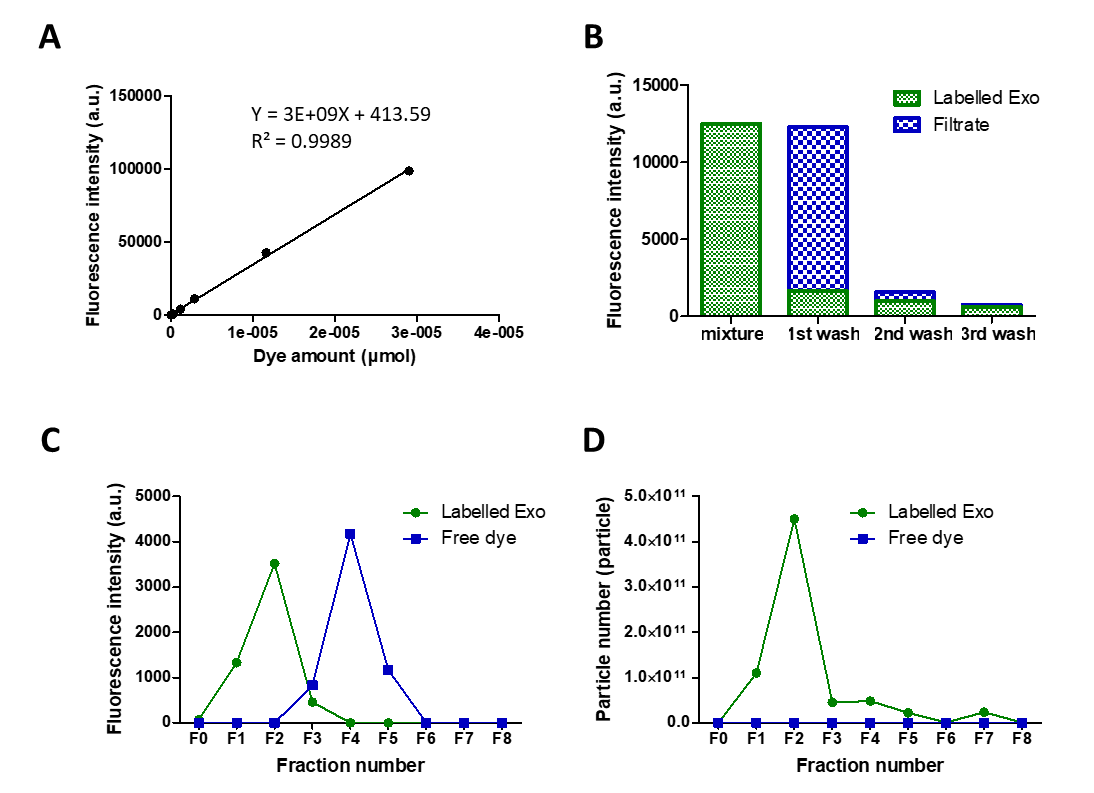


**Figure S4**. **Optimisation of purification method for removal of free dye.** (A) The calibration curve of free Alexa Fluor 488-azide dye. Fluorescence intensities were obtained by the fluorescence plate reader FLUOstar Omega plate reader at Ex/Em: 485/520 nm. (B) Fluorescence intensities of the upper (labelled exosomes) and lower (filtrate) fractions after 3 washes using ultrafiltration with NanoSep® for removal of free dye. (C) Elution profiles (fractions 0-8 i.e. F0-F8) of labelled exosomes or free dye using gel filtration with CL-2B columns for purification in terms of fluorescent intensity in each fraction. (D) the NTA values of all the fractions for both free dye and labelled exosomes using gel filtration with CL-2B columns for purification.

**
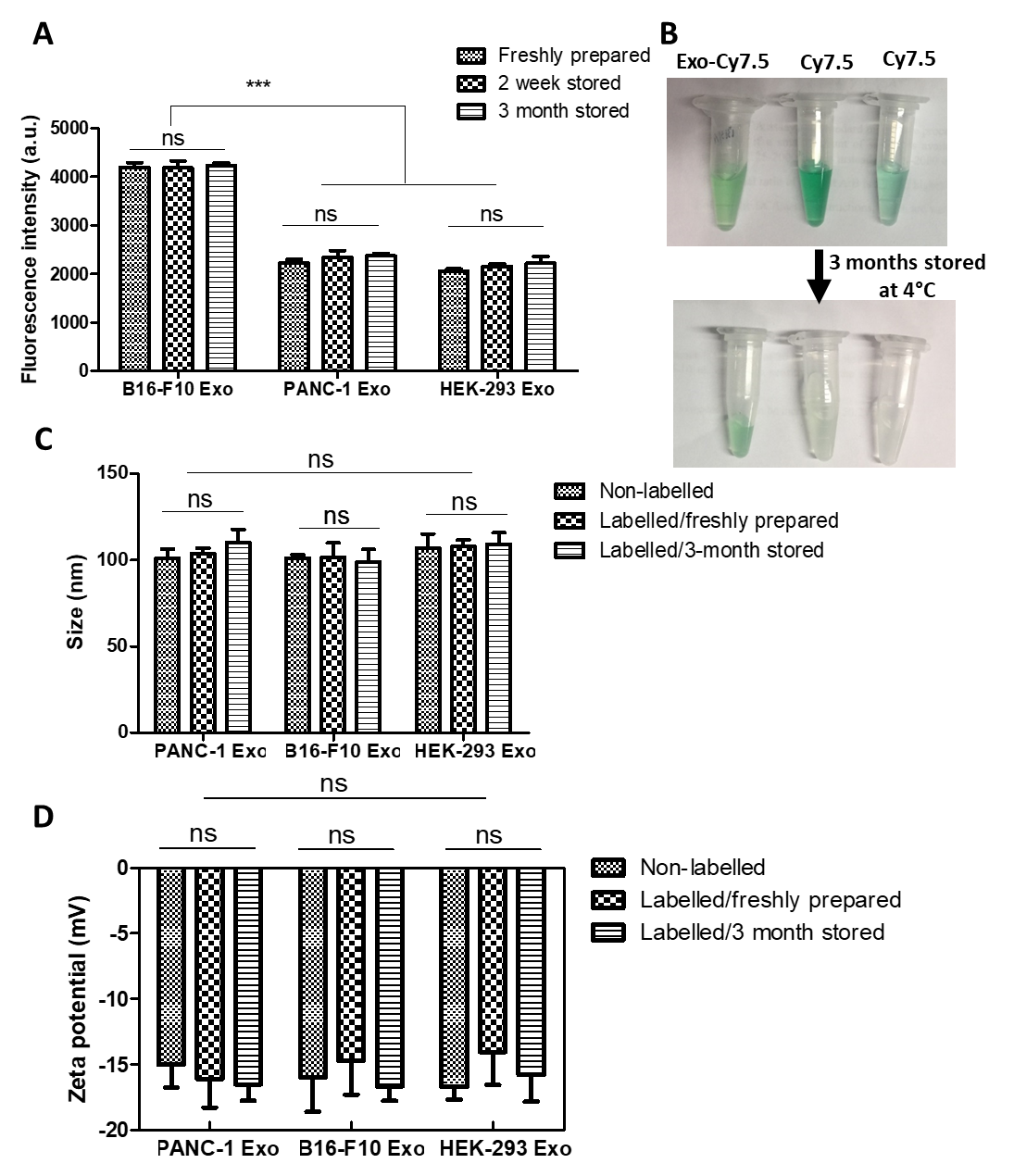
**

**Figure S5**. **Shelf-life stability of click chemistry-modified exosomes.** (A) The fluorescence intensities (FI) of exosomes either freshly labelled or stored for 2 weeks stored or 3 months at 4°C with protection from light. FI did not significantly change for all the three types of exosomes up to 3-month storage at 4°C (p > 0.05). (B) Representative images of freshly labelled and 3-month stored PANC-1 Exo and free dye (Cy7.5) in PBS, both protection from light. (C) Size and (D) zeta potential characterizations of labelled exosomes by ZetaSizer. All measurements were done in triplicate and values are displayed as mean ± SD. Statistical analysis were conducted on all samples (*** p<0.001).

**
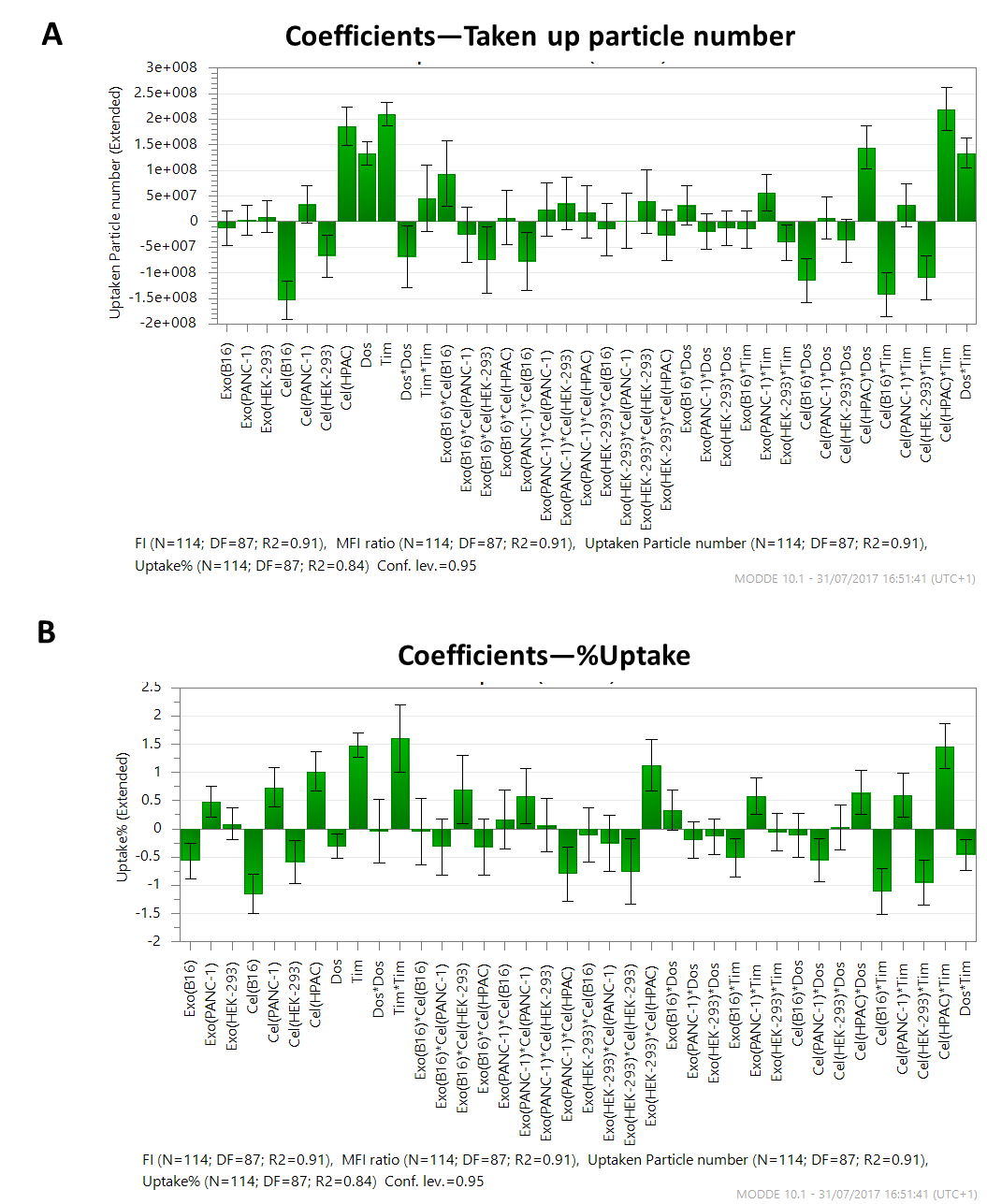
**

**Figure S6**. **Design of Experiment (DoE) modelling results.** Three types of exosomes (PANC-1, B16-F10, HEK-293 Exo), four cell lines (PANC-1, B16-F10, HEK-293, and HPAC cells), and other two factors (time and dose) were the inputs for modelling. Coefficients were calculated and plotted using MODDE 10.1 (Umetrics) software, which showed the coefficient denoting the significance of the effect of each factor or combination on Taken-up particle number (A) and %Uptake (B). Exo= exosomes, Cel = cell, Dos = dose, Tim = time.

**a.**

**Brightfield**

**AF488**

**b.**

**c.**

**d.**

**Figure S7**. **Representative images of PANC-1 cells under the Imagestream imaging flow cytometry.** Each cell is simultaneously imaged under a bright field and a fluorescence (AF488) channel. Incubation conditions: PANC-1 Exo, 12 h, 2.0 × 10^10^ particles.

**Figure S8. In *vitro* cytotoxicity assessment of labelled Exo in PANC-1 cells.** Cell viability was determined by MTT assay after 24 h treatment with three types of labelled Exo at a concentration of 2.0 × 10^10^ particles. Cells treated with 10% DMSO were used as positive control for cytotoxicity. Cell viability is expressed as percentage of untreated cells.

**Figure S9**. **Panel of typical images from the Imagestream Cytometer for PANC-1 cells.** Each cell is simultaneously imaged in a bright field and a fluorescence (AF488) channel. Incubation conditions: PANC-1 Exo, 12 h, 2.0 × 10^10^ particle/mL.

**
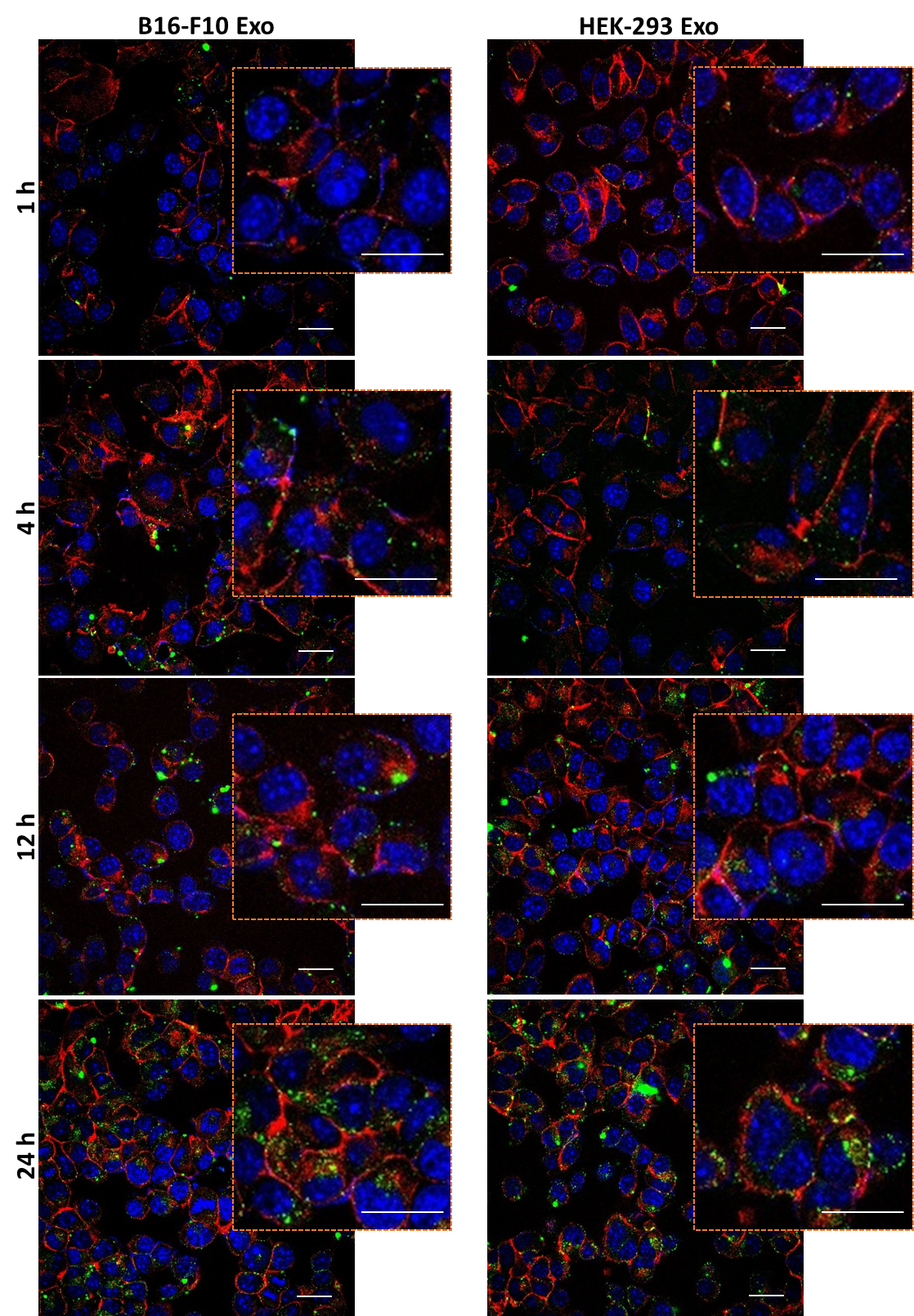
**

**Figure S9**. **Spatial distribution of B16-F10 and HEK-293 Exo taken up by PANC-1 cells obtained by Confocal Laser Scanning Microscopy (CLSM)**. The exosomes were fluorescently labelled with AF488 dye (green). Nuclei were counter-stained with DAPI (blue) and F-actin was stained with AF568 phalloidin (red). Orange signals indicate overlap of red and green signals. Scale bar: 20 µm.


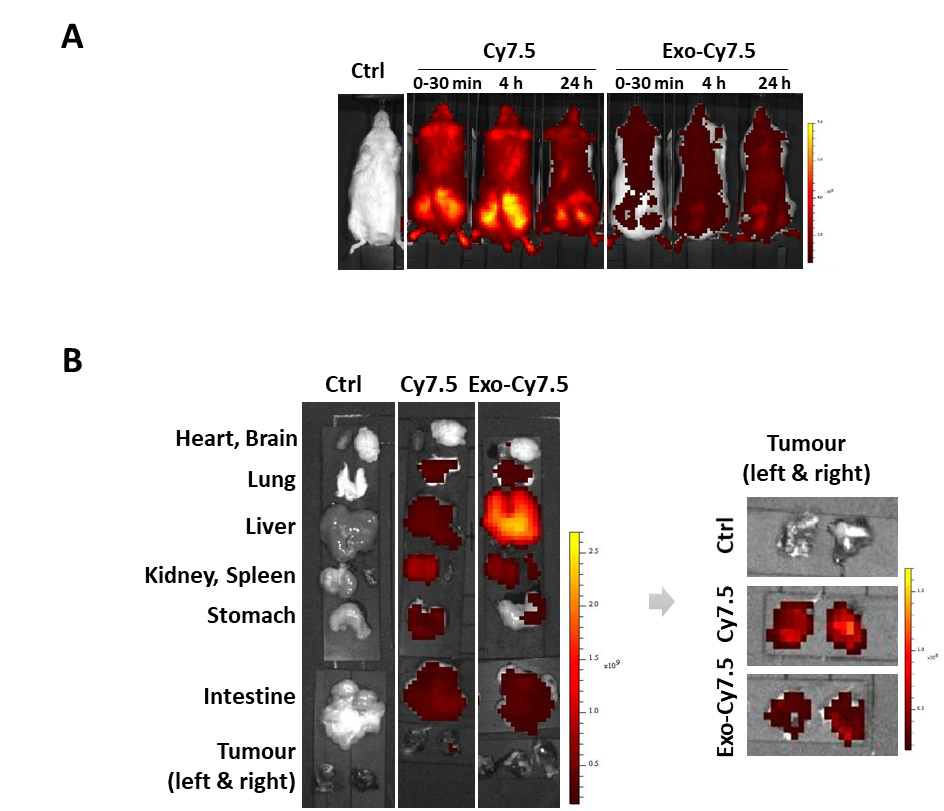


**Figure S10. *In vivo* organ biodistribution profile of PANC-1 Exo in B16-F10 tumour-bearing NSG mice.** Mice were inoculated subcutaneously with B16-F10 cells on both flanks (bilateral inoculation). Animals were intravenously injected with 200 µL of either 8 × 10^11^ PANC-1 Exo-Cy7.5 containing approximately 8.3 pmol of Cy7.5 or free Cy7.5 only (8.3 pmol) in saline were used as controls. Animals were culled at 24 h post-injection and the organs were excised for analysis. (A) Whole body live imaging. (B) *Ex vivo* imaging.

**Table S1. Shelf-life stability of fluorescently labelled exosomes (B16-F10, PANC-1, and HEK-293) prepared freshly or stored for 3 months at 4°C.**

| **Exosome** | **Labelled/Freshly prepared** | | | **Labelled/3-month stored** | | |
| --- | --- | --- | --- | --- | --- | --- |
|  | **Concentration (particle/mL)^a,c,d^** | **Size (nm) ^a,c^** | **Zeta potential (mV) ^b,c^** | **Concentration (particle/mL)^a,c,d^** | **Size (nm) ^a,c^** | **Zeta potential (mV) ^b,c^** |
| PANC-1 | 5.6 ± 0.3 × 10^11^ | 103.7 ± 3.2 | -16.1 ± 2.2 | 5.7 ± 0.2 × 10^11^ | 110.2 ± 7.6 | -16.5 ± 1.3 |
| B16-F10 | 8.0 ± 0.2 × 10^11^ ** | 101.6 ± 8.3 | -14.7 ± 2.6 | 8.2 ± 0.9 × 10^11^** | 99.0 ± 7.2 | -16.7 ± 1.1 |
| HEK-293 | 5.4 ± 0.2 × 10^11^ | 107.8 ± 3.7 | -14.0 ± 2.5 | 5.3 ± 0.7 × 10^11^ | 109.0 ± 6.8 | -15.6 ± 2.1 |

^a^ Values were obtained with NTA. Exosomes (1000 µL in total) were stored in 1.5 mL Eppendorf tubes, respectively.

^b^ Samples were diluted 10 times with water before measurement by Zetasizer Nano ZS at 25°C.

^C^ Values were expressed as mean ± SD.

^d^ One-way ANOVA was performed to evaluate the difference between the experimental data where statistical difference is noted when p-value < 0.05. **p < 0.01.

**Table S2. Summary of significant factors along with scaled and centred coefficients.**

| **Response** | **Factor** | **Coefficient** |
| --- | --- | --- |
| Taken-up particle number | **Cell type** |  |
|  | Cel(B16) | -1.53E+08 |
|  | Cel(HEK-293) | -6.71E+07 |
|  | Cel(HPAC) | 1.86E+08 |
|  | **Dose** | 1.34E+08 |
|  | **Time** | 2.10E+08 |
|  | **Exosome type*Cell type** |  |
|  | Exo(B16)*Cel(B16) | 9.35E+07 |
|  | Exo(B16)*Cel(HEK-293) | -7.55E+07 |
|  | Exo(PANC-1)*Cel(B16) | -7.81E+07 |
|  | **Exosome type*Time** |  |
|  | Exo(PANC-1)*Tim | 5.64E+07 |
|  | Exo(HEK-293)*Tim | -4.12E+07 |
|  | **Cell type*Dose** |  |
|  | Cel(B16)*Dos | -1.15E+08 |
|  | Cel(HPAC)*Dos | 1.45E+08 |
|  | **Cell type*Time** |  |
|  | Cel(B16)*Tim | -1.42E+08 |
|  | Cel(HEK-293)*Tim | -1.10E+08 |
|  | Cel(HPAC)*Tim | 2.20E+08 |
| Uptake percentage | **Exosome type** |  |
|  | Exo(B16) | -0.568193 |
|  | Exo(PANC-1) | 0.479869 |
|  | **Cell type** |  |
|  | Cel(B16) | -1.15934 |
|  | Cel(PANC-1) | 0.73321 |
|  | Cel(HEK-293) | -0.589227 |
|  | Cel(HPAC) | 1.01535 |
|  | **Dose** | -0.311848 |
|  | **Time** | 1.47839 |
|  | **Exosome type*Cell type** |  |
|  | Exo(B16)*Cel(HEK-293) | 0.697987 |
|  | Exo(PANC-1)*Cel(PANC-1) | 0.580716 |
|  | Exo(PANC-1)*Cel(HPAC) | -0.803973 |
|  | Exo(HEK-293)*Cel(HEK-293) | -0.758884 |
|  | Exo(HEK-293)*Cel(HPAC) | 1.12919 |
|  | **Exosome type*Time** |  |
|  | Exo(B16)*Tim | -0.519144 |
|  | Exo(PANC-1)*Tim | 0.580692 |
|  | **Cell type*Dose** |  |
|  | Cel(PANC-1)*Dos | -0.556211 |
|  | Cel(HPAC)*Dos | 0.64192 |
|  | **Cell type*Time** |  |
|  | Cel(B16)*Tim | -1.10828 |
|  | Cel(PANC-1)*Tim | 0.595516 |
|  | Cel(HEK-293)*Tim | -0.953195 |
|  | Cel(HPAC)*Tim | 1.46596 |
|  | **Dose*Time** | -0.458292 |

Asterisk (*) represents the interaction between two factors. Coefficient values reveal how much that factor can affect responses. Abbreviations: Exo= exosomes, Cel= cell, Dos= dose, Tim= time.
